# The glucose sensor NSUN2-m^5^C modification regulates tumor-immune glucose metabolism reprogramming to drive hepatocellular carcinoma evolution

**DOI:** 10.7150/ijbs.115610

**Published:** 2025-07-11

**Authors:** Jing He, Boqiang Liu, Weijun Zhao, Hao Shen, Yi Wang, Weiqi Li, Chenqi Jin, Yifan Wang, Xiujun Cai, Liang Shi

**Affiliations:** 1 Zhejiang Provincial Key Laboratory of Laparoscopic Technology, Sir Run Run Shaw Hospital, Zhejiang University, Hangzhou, 310016, China.; 2 Department of General Surgery, Sir Run Run Shaw Hospital, School of Medicine, Zhejiang University, Hangzhou, 310016, China.; 3 Zhejiang Minimal Invasive Diagnosis and Treatment Technology Research Center of Severe Hepatobiliary Disease, Zhejiang University, Hangzhou, 310016, China.; 4 Zhejiang Research and Development Engineering Laboratory of Minimally Invasive Technology and Equipment, Zhejiang University, Hangzhou, 310016, China.; 5 Zhejiang University Cancer Center, Zhejiang University, Hangzhou, 310016, China.

**Keywords:** tumor evolution, metabolic reprogramming, tumor immune microenvironment, single-cell sequencing, 5-methylcytosine modification

## Abstract

Tumor heterogeneity and the dynamic evolution of tumor immune microenvironment (TIME) contribute to therapeutic resistance and poor clinical prognosis. To elucidate this mechanism, we first established a murine tumor evolution model (TEM) and systematically identified evolutionary core genes demonstrating progressive alterations during evolution. Subsequently, we developed a single-cell TEM through integrative analysis of hepatocellular carcinoma (HCC) clinical specimens (n=10) with external cohorts (n=11), enabling dynamic characterization of tumor-immune interactions during evolution, while addressing ethical challenges associated with obtaining tumor tissues from multiple stages in a single patient. Through TEMs analyses, we identified a contrasting glucose metabolism pattern between malignant cells and CD8^+^ T cells during tumor evolution. Mechanistically, glucose metabolic dominance triggers NSUN2 upregulation in tumor cells, where this functional RNA methyltransferase stabilizes key glycolytic transcripts (GLUT1, HK2, PFKM) through mRNA methylation. The NSUN2-mediated GLUT1 stabilization enhances the competitive advantage of tumor cells in glucose acquisition, creating a positive feedback loop that accelerates malignancy and exacerbates CD8^+^ T cell dysfunction. Building on these insights, we designed a dual-targeting strategy combining GLUT1/NSUN2 axis inhibitor WZB117 with PD-L1 blockade, which synergistically suppressed tumor evolution and reversed immunosuppression in preclinical models, suggesting a novel synergistic therapeutic strategy for treatment-resistant HCC.

## Introduction

Hepatocellular carcinoma (HCC) ranks as the sixth most common cancer worldwide and is the second leading cause of cancer-related deaths 1. Due to its subtle symptoms and aggressive progression, the majority of patients are diagnosed at advanced stages 2. Immunotherapy has emerged as one of the most promising strategies for combating tumors 3. However, only 15-20% of HCC patients respond to immunotherapy, with limited improvements in overall survival observed in most cases 4, 5. A key challenge lies in the remarkable plasticity and adaptability of tumors within the tumor immune microenvironment (TIME) and under various therapeutic pressures. This phenomenon, known as tumor evolution, significantly undermines the efficacy of single-agent treatments 6-8.

Within the TIME, intricate interactions and regulatory relationships occur among its diverse components 9. A key aspect of these interactions is the intense metabolic competition between malignant cells and immune cells for shared nutrients, which plays a crucial role in shaping tumor initiation and progression 10, 11. For instance, glutamine competition has been shown to drive the immunosuppressive reprogramming of intratumoral GPR109A^+^ myeloid cells in HCC, facilitating immune evasion and promoting tumor growth 12. Similarly, the Warburg effect drives tumor cells within the TIME to consume large amounts of glucose for rapid proliferation. This intense metabolic competition deprives T cells, which also rely on glucose for activation and function, potentially impairing CAR-T cell efficacy and promoting immune evasion 13. Emerging evidence reveals that metabolic reprogramming in the TIME orchestrates antitumor immunity through multilayered regulatory mechanisms. For example, succinyl-CoA, traditionally viewed as merely a metabolic intermediate, actively regulates immune checkpoints to limit tumor immune evasion 14. Furthermore, tumor cells can directly disrupt T cell-mediated immune surveillance through transfer of mitochondria containing pathogenic mtDNA mutations 15. These findings reveal the multifaceted nature of metabolic crosstalk in the TIME, while the specific role of metabolic-immune signaling in HCC evolution warrants further investigation.

In many cases, nutrients or metabolites within the TIME exert their effects by acting as direct substrates for biochemical reactions 16, 17. Methionine, through its metabolite S-adenosylmethionine (SAM), contributes to DNA and protein methylation while activating tumor-associated macrophages (TAMs) to suppress tumor progression 18. Glucose fuels the glycolysis pathway, where it is broken down into pyruvate while generating ATP and NADH. This pathway is preferentially utilized by tumor cells to support their rapid growth and metabolic demands 19. Interestingly, recent studies have revealed that leucine can directly activate mTORC1 signaling by binding to its specific receptor, the SAR1B protein 20. Furthermore, glucose has been found to interact with DDX21, promoting the dissociation of DDX21 dimers and thereby regulating mRNA splicing and tissue differentiation 21. These findings suggest that beyond their conventional roles in metabolic pathways, nutrients can act as independent signaling molecules, mediating diverse cellular processes crucial for tumor development and cellular adaptation within the TIME.

According to our tumor evolution model (TEM), the competition for glucose between malignant cells and CD8^+^ T cells is a critical driver of HCC evolution. Excessive glucose uptake by malignant cells markedly increases NSUN2 expression, which in turn stabilizes the mRNA of GLUT1, HK2, and PFKM in an m^5^C-dependent manner, facilitating metabolic reprogramming. This process establishes a positive feedback loop, wherein the upregulation of GLUT1 further enhances the glucose uptake capacity of malignant cells, promoting tumor evolution while suppressing the anti-tumor activity of CD8^+^ T cells. Furthermore, we propose a novel immunometabolic therapy targeting the glucose-competition/NSUN2 axis using WZB117 in combination with anti-PD-L1 treatment. This strategy not only overcomes resistance to immune checkpoint blockade but also synergistically attenuates tumor evolution, thereby bridging the interplay between immunometabolic therapy and tumor evolution.

## Materials and Methods

### Patients

A cohort of 10 HCC patients was established, who underwent surgical treatment at the Sir Run Run Shaw Hospital. All patients signing Informed Consent before surgery for the use of their tissues for scientific research. This study conformed to the principles of the Declaration of Helsinki and was approved by the Ethics Committee of Sir Run Run Shaw Hospital (SRRSHLS2022Y0312).

### Animal studies

C57BL/6 mice were purchased from Qizhen Experimental Animal Technology (Hangzhou, CN). C57BL/6JGpt-*H11^em1Cin(Tcra&Tcrb)^*/Gpt (OT-1) mice were purchased from GemPharmatech (Nanjing, CN). In the *in vivo* syngeneic tumorigenesis assays, 2 × 10⁶ cells suspended in 50 μL were subcutaneously injected into 4-week-old male C57BL/6 mice (n = 6 per group). For experiments involving glucose supplementation (15% glucose in drinking water), the injected cell number was uniformly reduced to 2 × 10⁵ cells (also in 50 μL) for all mice in that experiment to minimize tumor burden and comply with ethical guidelines, as suggested by preliminary data. The dosing regimen for the anti-mouse PD-L1 antibody (Clone 10F.9G2) or rat IgG2b isotype control antibodies was 10 mg/kg administered intraperitoneally twice a week 22. For WZB117 (dissolved in PBS/DMSO solution) or the PBS/DMSO vehicle, daily intraperitoneal injections at a dose of 10 mg/kg were employed 23. Tumor dimensions were measured using calipers to determine length and width, and tumor volume was calculated using the formula: V = (Length × Width × Width)/2. These mice were singly-housed with environmental enrichment. All animals were maintained on a 12-hour light/dark cycle, in a humidity- and temperature-controlled room with water and food available ad libitum. All mouse procedures were conducted under the guidelines and the institutional animal care protocol approved by the Experimental Animal Committee at Zhejiang University (ZJU20240983).

### Cell culture

Hepa1-6, Huh7, HCCLM3, and Jurkat cell lines were maintained under physiological glucose conditions (5.5 mM D-glucose) in their respective media supplemented with 10% fetal bovine serum at 37 °C with 5% CO_2_. Hepa1-6 and HCCLM3 cells were cultured in Dulbecco's Modified Eagle Medium (DMEM). Huh7 cells were maintained in Minimum Essential Medium (MEM). In the process of T cell-related experiments, T cells were cultured in the RPMI-1640 medium supplemented with 25 µL/mL ImmunoCult™ CD3/CD28 T cell activator and 10 ng/mL recombinant interleukin-2 (rIL-2).

### Reagents (e.g. antibodies, drugs, proteins, primers, vectors etc.)

Detail of these reagents were included in Supplementary Materials.

### Murine tumor evolution model

To establish the orthotopic syngeneic model, 2 × 10^6^ Hepa1-6 cells were resuspended in 50 μL of serum-free DMEM; 50 μL of this mixture was injected into the left lobe of the liver in C57BL/6 mice. Mice injected with Hepa1-6 cells were sacrificed at 2, 4, and 6 weeks post-injection, and samples were collected for subsequent analyses.

### Identification of evolutionary core genes

Differential expression analysis was performed using the DESeq2 package, and differentially expressed genes (DEGs) were identified based on an adjusted *p-value* < 0.05 (with Benjamini-Hochberg correction) and |log_2_(Fold Change)| ≥ 1. Weighted gene co-expression network analysis (WGCNA) was conducted using UMI counts, with a soft-thresholding power of 3 determined by the pickSoftThreshold function 24. Modules were detected using the "tree" method with a minimum size of 10 genes and the deepSplit parameter enabled. By intersecting DEGs with characteristic WGCNA modules, candidate features were refined and further selected using support vector machine—recursive feature elimination (SVM-RFE) 25. The svmRFE function incorporated feature elimination into a ten-fold cross-validation framework, with k = 10 controlling the number of features removed per iteration. We performed fuzzy c-means clustering using the R package Mfuzz to identify genes with similar expression patterns during tumor evolution 26. For each time point, expression values were averaged across biological replicates and standardized (mean = 0, standard deviation = 1) to facilitate clustering in Euclidean space. Soft clustering assigned membership scores (0-1) to each gene across four clusters, and core genes were identified using a membership score cutoff of 0.5, yielding 21-67 genes per cluster.

### Survival analysis

Survival analysis was conducted using the R packages survival (v3.5.5) and survminer (v0.4.9). Patients from the TCGA and ICGC Japan cohorts were stratified into high- and low-risk groups based on the expression levels of evolutionary core genes. Kaplan-Meier survival curves were generated using the ggsurvplot function and compared using the log-rank test. Additionally, associations between clinical variables and risk groups were assessed using one-way ANOVA.

### Enrichment analysis

Gene set variation analysis (GSVA) was performed using the R package GSVA. This non-parametric and unsupervised method transforms a gene-by-sample expression matrix into a gene set-by-sample matrix, calculating enrichment scores for each sample and pathway. Overrepresented Gene Ontology (GO) terms were identified using the clusterProfiler package (v4.2.2) 27. Adjusted *p-values* were computed using the Benjamini-Hochberg (BH) method, with a significance threshold of < 0.05.

### Single-nucleus RNA-sequencing data collection and quality control

Nuclei were isolated from tumor samples of 10 HCC patients using Nuclei EZ Lysis Buffer supplemented with protease and RNase inhibitors. cDNA synthesis and library preparation were performed following the manufacturer's standard protocol, and sequencing was conducted on an Illumina NovaSeq 6000 platform (paired-end, 150 bp reads) by LC-Bio Technology Co. Ltd. (Hangzhou, China), targeting a minimum of 20,000 reads per nucleus. Raw FASTQ files were processed using Cell Ranger (v7.1.0, 10X Genomics), aligned to the GRCh38 human reference genome (GENCODE v32 / Ensembl 98), and gene-by-cell count matrices were generated for downstream analysis in R. Quality control was conducted using the `runScStatistics` function from the **scCancer** package (v2.2.1). Nuclei were retained only if they had >400 UMIs, >500 detected genes, and <4% mitochondrial gene expression. In addition, publicly available single-cell RNA-seq data of HCC tumors (GEO: GSE151530) were included for comparative and integrative analyses. To correct for batch effects between snRNA-seq data and external scRNA-seq data, we employed the LIGER package using integrative non-negative matrix factorization (iNMF) with 30 factors (k = 30) and a regularization parameter λ = 5, followed by joint clustering and quantile normalization.

### Construction of tumor evolution model (TEM)

Highly variable genes (HVGs) were identified using the Seurat FindVariableFeatures function with variance-stabilizing transformation (selection.method = "vst"), and the top 2,000 most variable features were selected for downstream analysis. Principal component analysis (PCA) was performed on these HVGs using the RunPCA function. For dimensionality reduction and visualization, the top 20 principal components were used to construct a shared nearest neighbor (SNN) graph via the FindNeighbors function, followed by cell clustering using FindClusters at a resolution of 0.8. Cell types were annotated based on canonical marker gene expression, resulting in the identification of distinct clusters including malignant cells, epithelial cells, endothelial cells, hepatic stellate cells (HSCs), macrophages, B cells, CD8^+^ T cells, CD4^+^ T cells, and NK cells. To build the TEM, malignant cells were further stratified into three evolutionary stages ("early," "mid," and "advanced") using k-means clustering (k = 3), with the optimal number of clusters determined by the maximum silhouette coefficient. Finally, unsupervised hierarchical clustering was applied to the samples to characterize the evolutionary phases of the TIME.

### Trajectory analysis

To reconstruct cellular trajectories and order cells along a pseudotemporal axis, we used the Slingshot package, a widely adopted tool for lineage inference 28. Slingshot leverages pre-defined cell clusters to construct lineage relationships by generating a minimum spanning tree (MST). Applying this approach enabled us to delineate the dynamic progression of cellular states within the dataset, thereby supporting a comprehensive analysis of HCC evolution.

### Metabolism analysis

Signature score of metabolism pathways in single-cell resolution was quantified by scMetabolism (v0.2.1) against REACTOME gene sets 29.

### Tissue preparation and immunohistochemistry

Formalin-fixed, paraffin-embedded tumor tissues were sectioned at 4 μm. Following deparaffinization and rehydration, antigen retrieval was performed by heating the sections at 95 °C in citrate buffer (pH 6.0), followed by treatment with hydrogen peroxide to block endogenous peroxidase activity. To reduce nonspecific antibody binding, sections were incubated in blocking buffer (10% normal goat serum in PBST) at room temperature for 1 hour. Primary antibodies were applied overnight at 4 °C, including rabbit anti-Ki-67 (1:400), anti-N-cadherin (1:5,000), anti-VEGFα (1:500), and anti-CD8 (1:2,000). Sections were then incubated with secondary antibodies for 2 hours at room temperature. Finally, slides were counterstained with acid hematoxylin solution (12 min) and Scott's bluing reagent (5 min), dehydrated through a graded ethanol series to 100%, cleared in xylene (5 min), and coverslipped using Tissue-Tek Glas mounting medium.

### Glucose starvation and restoration

For glucose deprivation, cells were washed twice with PBS and then cultured in glucose-free DMEM or RPMI-1640 supplemented with dialyzed FBS for the indicated duration. Glucose restoration was performed by adding DMEM containing glucose, 2-deoxy-D-glucose (2-DG), 2-NBDG, BAY-876, or WZB117 for the specified time period.

### Cell counting kit-8 assay

Cell proliferation was assessed using the Cell Counting Kit-8 (CCK-8) assay. Briefly, cells were seeded in 96-well plates at a density of 1 × 10⁴ cells per well. At 24, 48, and 72 hours, 10 μL of CCK-8 reagent was added to each well containing 100 μL of medium and incubated for 1 hour at 37 °C in a 5% CO_2_ atmosphere. Absorbance at 450 nm was measured using a spectrophotometer (Thermo, Grand Island, NY, USA) every 24 hours over a period of 3 days to generate cell growth curves, following the manufacturer's instructions.

### Cell migration assay

For the transwell migration assay, HCC cells were seeded into the upper chambers of transwell inserts in serum-free medium at a density of 0.2-1 × 10⁵ cells per well. The lower chambers were filled with culture medium containing 10% FBS as a chemoattractant. Cells were incubated at 37 °C for 24 hours. After incubation, cells that had migrated to the lower surface of the membrane were fixed with 4% paraformaldehyde and stained with 0.1% crystal violet for visualization.

### Colony formation assay

For the colony formation assay, 500 cells per well were seeded into 24-well plates and cultured for 14 days. Cells were then washed twice with PBS, fixed with 4% paraformaldehyde for 15 min, and stained with 0.5% crystal violet for 30 min at room temperature.

### Extracellular acidification rate (ECAR) assay

Extracellular acidification rate (ECAR) was measured using the XF96 Extracellular Flux Analyzer (Seahorse Bioscience). Malignant cells were seeded at a density of 1.0 × 10⁵ cells per well in XF96 plates and incubated overnight. OT-1 CD8^+^ T cells or Jurkat cells were seeded at 1.0 × 10⁴ cells per well in XF96 plates pre-coated with Cell-Tak adhesive. Plates were briefly centrifuged to immobilize the cells. One hour prior to the assay, the culture medium was replaced with XF assay medium. The XF Glycolysis Stress Test Kit was used to assess glycolytic function. Glucose, oligomycin, and 2-DG were diluted in XF assay medium to final concentrations of 10 mM, 1 μM, and 50 mM, respectively, and loaded into the reagent cartridge. ECAR was measured according to the manufacturer's instructions.

### Quantitative real-time PCR

Total RNA was extracted using TRIzol reagent (Invitrogen). One microgram of total RNA was reverse-transcribed using SuperScript III reverse transcriptase (Invitrogen). Quantitative real-time PCR (RT-qPCR) was performed on a Bio-Rad CFX96 system (Bio-Rad) using SYBR Green Master Mix to quantify target gene expression. Expression levels were normalized to β-actin (ACTB) mRNA.

### Glucose Uptake (2-NDBG incorporation) assays

Cells were washed twice with PBS for 5 min each. Following washing, 200 μM 2-NBDG was added to the cell suspension and incubated for 1 h at 37 °C. After incubation, cells were trypsinized, collected by centrifugation, and the supernatant was discarded. Cells were then washed twice more with PBS for 5 min each, resuspended in PBS, and analyzed by flow cytometry to evaluate 2-NBDG incorporation as a quantitative measure of glucose uptake.

### Intracellular cytokine production analysis

Primary CD8^+^ T cells were cultured in RPMI-1640 medium supplemented with 10% FBS, 10 ng/mL recombinant IL-2, and 50 μM β-mercaptoethanol. OVA-specific TCR transgenic OT-1 T cells were isolated from OT-1 mice and activated with the OVA-derived peptide SIINFEKL for 5 days. After activation, cells were washed twice with PBS for 5 min each and resuspended in PBS. Fixable Viability Stain (1:100) was added, and cells were incubated on ice for 10 min. After washing with PBS, cells were stained with surface antibodies including CD45 (1:200), CD3 (1:200), and CD8 (1:200) at 4 °C for 30 min. Following surface staining, cells were fixed with Fixation Buffer for 20 min at room temperature in the dark, then permeabilized using Intracellular Staining Permeabilization Wash Buffer. After centrifugation and removal of the supernatant, cells were resuspended in the same buffer and stained with intracellular antibodies including GZMB (1:30), perforin (1:50), IFN-γ (1:100), and TNF-α (1:100) for 1 h at room temperature in the dark. After washing to remove unbound antibodies, cells were resuspended in PBS and analyzed by flow cytometry.

### *In vitro* cytotoxicity assay

OVA-specific TCR transgenic OT-1 T cells were isolated from OT-1 mice and activated using the OVA-derived peptide SIINFEKL. OVA-expressing Hepa1-6 cells (referred to as OVA-Hepa1-6) were generated via lentiviral transduction. Tumor cells were labeled with carboxyfluorescein succinimidyl ester (CFSE) according to the manufacturer's protocol and seeded at a density of 1 × 10⁵ cells per well. Effector cells (purified OT-1 T cells) were then added to each well, and co-cultures were incubated for 24 h at 37 °C in a humidified 5% CO_2_ incubator using medium containing 5.5 mM glucose. Following incubation, cells were stained with propidium iodide (PI) to identify dead cells. Samples were analyzed by flow cytometry. CFSE-positive tumor cells and PI-positive dead cells were quantified to determine the percentage of tumor cell lysis.



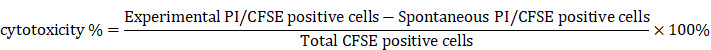



### Immunoblotting

Total proteins were extracted using RIPA lysis buffer. Protein samples were separated by sodium dodecyl sulfate-polyacrylamide gel electrophoresis (SDS-PAGE) and transferred onto a PVDF membrane. Membranes were incubated overnight at 4 °C with primary antibodies, followed by incubation with appropriate secondary antibodies at room temperature for 1 hour. Protein bands were visualized using enhanced chemiluminescence (ECL) reagents to detect antigen-antibody complexes.

### m^5^C dot blot assay

Equal amounts of RNA were spotted onto a positively charged nylon membrane (GE Healthcare). After UV crosslinking using a Stratalinker UV Crosslinker 1800 (254 nm, 3 minutes), the membrane was blocked with 5% non-fat milk in PBS containing 0.1% Tween-20 (PBST). The membrane was then incubated with a rabbit anti-m⁵C primary antibody (1:1000), followed by a peroxidase-conjugated AffiniPure goat anti-rabbit IgG (H+L) secondary antibody (1:2000). m⁵C RNA levels were detected using enhanced chemiluminescence (ECL) reagents. Equal RNA loading was confirmed by methylene blue staining.

### Methylated RNA immunoprecipitation (MeRIP) and RNA immunoprecipitation (RIP) assays

Real-time quantitative PCR (qPCR) was used to assess the relative abundance of selected mRNAs in m⁵C antibody immunoprecipitation (IP) and corresponding input samples. Total RNA was extracted using Trizol reagent (Invitrogen). A total of 500 ng RNA was reserved as input, while the remaining RNA was used for m⁵C-IP. For each IP reaction, 100 μg of RNA was diluted into 500 μL IP buffer (150 mM NaCl, 0.1% NP-40, 10 mM Tris, pH 7.4) supplemented with 100 U RNase inhibitor, and incubated with an anti-m⁵C antibody at 4 °C for 2 hours. BSA-coated Dynabeads Protein A were then added and rotated for an additional 2 hours at 4 °C. After four washes with IP buffer containing RNase inhibitors, the m⁵C-enriched RNA was eluted using elution buffer (5 mM Tris-HCl [pH 7.5], 1 mM EDTA, 0.05% SDS, and 4 μL Proteinase K [20 mg/mL]). Equal amounts of IP RNA and input RNA were used for cDNA synthesis. mRNA expression was determined by quantification cycle (Cq) values, and relative m⁵C enrichment for each gene was calculated by normalizing IP values to corresponding input values.

RIP assays for NSUN2-mRNA interactions were performed using an anti-NSUN2 antibody following the same procedure as described for MeRIP. Immunoprecipitated RNAs were subsequently analyzed by qPCR to identify NSUN2-bound transcripts.

### RNA decay assay

Cells were seeded in 6-well plates and incubated overnight at 37 °C. Subsequently, cells were treated with actinomycin D (5 μg/mL) for varying durations, after which RNA was extracted for analysis. mRNA levels were quantified by real-time quantitative PCR (qPCR). The half-lives of GLUT1, HK2, and PFKM mRNAs were normalized to β-actin, with expression levels at time zero (t = 0) set to 100%, consistent with previously published protocols 30.

### ATP measurement

Cellular ATP levels were measured using an ATP assay kit (Beyotime Biotechnology) following the manufacturer's instructions. Briefly, cell lysates were centrifuged at 12,000 × g for 10 min to remove debris, and the supernatant was mixed with the substrate solution. Luminescence was measured using a microplate luminometer with an integration time of 10 s per well.

### Quantification and statistical analysis

GraphPad Prism v.9.5.1 was used to calculate statistical significance. All experiments were independently performed at least three times. Results are presented as mean ± standard deviation (SD). Comparisons were conducted using paired or unpaired Student's t test, one-way ANOVA, or two-way ANOVA, as indicated in the respective figure legends.

## Results

### Identification of evolutionary core genes in murine tumor evolution model (TEM)

**Figure 1:**
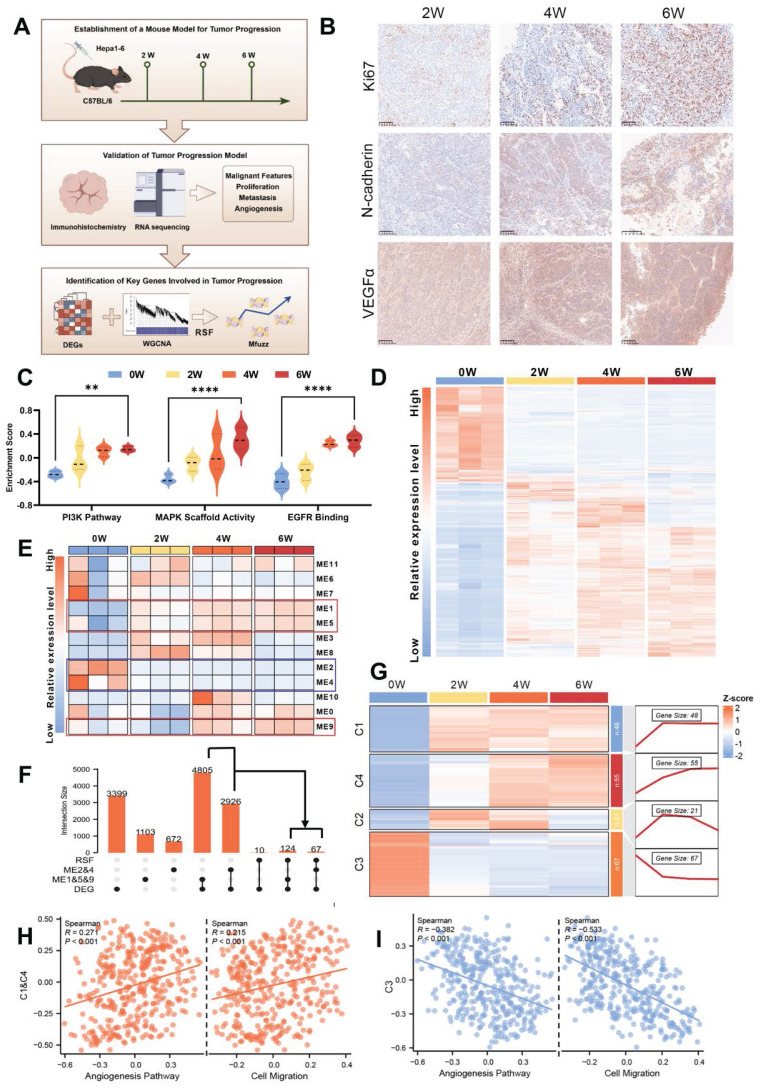
**Identification of evolutionary core genes in murine tumor evolution model (TEM). A.** Schematic diagram illustrating the workflow of study design and analysis. **B.** Immunohistochemical staining of Ki67, N-cadherin, and VEGFα in tumor tissues from an orthotopic hepatocellular carcinoma model established by the transplantation of Hepa1-6 cells into the livers of C57BL/6 mice (murine tumor evolution model, murine TEM). Tumor samples were collected at indicated time points post-transplantation, with brown signals indicating positive staining. **C.** Comparison of GSVA scores for pathways associated with tumor malignancy across distinct time points post-transplantation in murine TEM. Data are mean ± SD. *p < 0.05, **p < 0.01, ***p < 0.001, ****p < 0.0001, ns, non-significant by two-way ANOVA. **D.** Heatmap depicting the comparative gene expression profiles in tumor tissues harvested from the murine TEM at distinct time points post-transplantation, highlighting transcriptomic changes during tumor evolution. Differentially expressed genes (DEGs) were identified based on the criteria of p < 0.05 and |log_2_(Fold Change)| ≥ 1. **E.** Weighted Gene Co-Expression Network Analysis (WGCNA) identified a total of 12 merged modules. The heat map illustrates the expression level changes of co-expression modules across distinct time points post-transplantation in murine TEM. **F.** The intersection of DEGs with co-expression modules was further analyzed using Support Vector Machine-Recursive Feature Elimination (SVM-RFE) algorithm. The UpSet plot indicated this relationship. **G.** Genes clustered according to their expression patterns along tumor evolution, utilizing Mfuzz clustering analysis. **H and I.** Correlation between angiogenesis and cell migration GSVA enrichment scores with Mfuzz clusters: C1 & C4 (**H**), C3 (**I**) in the TCGA-LIHC cohort.

Research into tumor evolution is heavily constrained by clinical ethical limitations on acquiring tumor tissues at various stages from the same patient. To overcome this challenge, we developed a murine TEM through orthotopic implantation of Hepa1-6 cells into the livers of C57BL/6 mice, effectively replicating the *in situ* microenvironment characteristic of HCC. Tumor samples were collected every 14 days, and high-throughput transcriptome sequencing was conducted to characterize the dynamic changes occurring during tumor evolution (Fig. 1A).

Malignancy-associated biomarker levels showed a gradual elevation across time points (Fig. 1B). Simultaneously, Gene Set Variation Analysis (GSVA) revealed a marked upregulation of malignancy-related pathways as tumor evolution progressed (Fig. 1C), further supporting our model's reliability. And significant alterations in transcriptomic profiles were observed across tumor evolution, suggesting these dynamic changes could serve as robust indicators of distinct evolutionary stages while providing insights into underlying biological processes (Fig. 1D).

Then, Weighted Gene Co-expression Network Analysis (WGCNA) identified key regulatory modules associated with tumor evolution. Among them, the ME1, ME5, and ME9 modules were progressively upregulated, while the ME2 and ME4 modules showed consistent downregulation over time (Fig. 1E and Fig. S1A). To refine this analysis, we employed the Support Vector Machine-Recursive Feature Elimination (SVM-RFE) algorithm, which identified evolutionary signature genes by iteratively ranking features based on their temporal relevance in the model (Fig. 1F). These genes were subsequently grouped into four distinct clusters (C1-C4) using fuzzy c-means clustering analysis (Mfuzz), which revealed dynamic expression patterns throughout tumor evolution. Specifically, genes in clusters C1 and C4 exhibited a gradual increase in expression, while those in cluster C3 demonstrated progressive downregulation from the second to the sixth week after orthotopic implantation (Fig. 1G). Based on these trends, we propose that genes in clusters C1, C3 and C4 represent evolutionary core genes (Supplementary Table 1), as their dynamic expression profiles likely reflect critical molecular mechanisms underlying tumor evolution.

Crucially, the key genes identified through the murine TEM were strongly associated with tumor evolution in human HCC. Specifically, these genes correlated significantly with various aspects of tumor malignancy, including tumor migration and angiogenesis (Fig. 1H and I). Furthermore, they showed strong associations with TNM staging (Fig. S1B and C) and patient prognosis (Fig. S1D and E). Taken together, these findings underscore the potential of the evolutionary core genes identified through the murine TEM as a foundation for exploring critical molecular mechanisms of tumor evolution and their applicability in advancing research on HCC.

### Glucose metabolic reprogramming is a crucial event during HCC evolution

**Figure 2:**
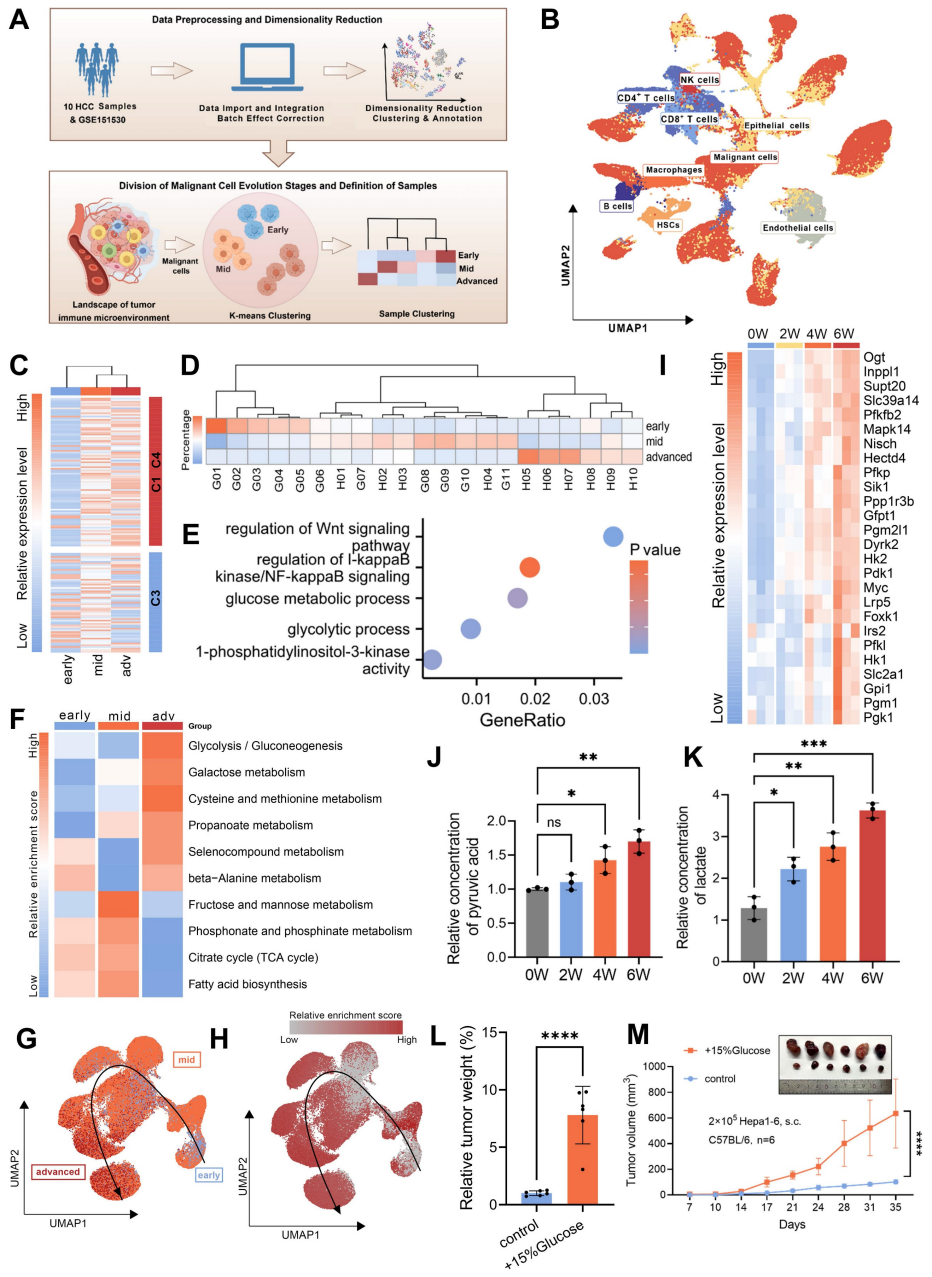
**Glucose metabolic reprogramming is a crucial event during HCC evolution. A.** Schematic diagram illustrating the workflow and study design for the TEM at single-cell resolution, including analytical strategies implemented in this research. **B.** Uniform Manifold Approximation and Projection (UMAP) plot showing cell type annotations within the HCC TIME, with corresponding color codes. **C.** Heatmap displaying the clustering of malignant cells based on the expression levels of evolutionary core genes in clusters C1 & C4 and C3. **D.** Heatmap presenting the clustering of HCC patient samples based on the proportions of tumor cells from different evolutionary stages (early, mid, and advanced) identified by the single-cell TEM. Samples labeled with “H” represent patients from the HCC cohort (n = 10), and those labeled with “G” are from the GEO dataset GSE151530 (n = 11). **E.** Gene Ontology (GO) enrichment analysis of characteristic genes associated with advanced stage malignant cells. **F.** Heatmap illustrating GSVA scores of metabolism-related pathways in malignant cells across early, mid, and advanced stages of HCC evolution. **G.** UMAP plot depicting the reconstructed evolutionary trajectory of malignant cells using the Slingshot algorithm. Cells from early, mid, and advanced stages are classified based on the TEM at single-cell resolution. **H.** UMAP plot demonstrating the GSVA scores for the glycolysis pathway along the Slingshot-inferred trajectory of malignant cells. **I.** Heatmap showing the expression levels of glycolysis pathway genes during HCC evolution in the murine TEM. **J and K.** The content of pyruvic acid (**J**) and lactate (**K**) in tumor cells or tissues derived from murine TEM at the indicated time after transplantation. **L and M.** Tumor weight (**L**) and volumes (**M**) were measured in an *in vivo* syngeneic tumorigenesis assay by subcutaneously injecting Hepa1-6 cells into C57BL/6 mice. The experimental group received drinking water supplemented with 15% glucose, while the control group received regular drinking water (n = 6 animals/group). Data are mean ± SD. *p < 0.05, **p < 0.01, ***p < 0.001, ****p < 0.0001, ns, non-significant by Student's t test (L and M) or by two-way ANOVA (J and K).

Having identified evolutionary core genes through transcriptomic analyses of the murine TEM, we next turned to single-nucleus RNA sequencing (snRNA-seq) to investigate the complex cellular dynamics and molecular pathways within the tumor immune microenvironment (TIME) that define tumor evolution. To enhance the robustness of our analysis, we integrated snRNA-seq data from an HCC patient cohort (n = 10) with an external publicly available dataset from the GEO database (accession GSE151530; n = 11). Prior to integration, rigorous quality control was performed, yielding a high-confidence combined dataset comprising 78,430 cells and 37,990 genes. To mitigate batch effects stemming from technical and cohort differences, we employed Liger for batch effect correction (Fig. 2A and Fig. S2A-B). Then we classified 9 major cell types across the dataset (Fig. 2B and Fig. S2C). K-means unsupervised clustering was employed to define the evolutionary stages of malignant cells, focusing specifically on the evolutionary core genes. This approach established a single-cell TEM, providing a foundational framework for understanding how dynamic changes within TIME (Fig. 2C). Building upon this analysis, we stratified HCC patient samples according to the dominant malignant cell evolutionary subpopulation identified in each sample. This patient-specific stratification revealed distinct patterns of TIME remodeling during evolution, enabling its classification into “early,” “mid,” and “advanced” evolutionary stages (Fig. 2D). These classifications offer valuable insights into the progressive interactions between malignant cells and their surrounding immune landscape, emphasizing a timeline of co-evolutionary dynamics.

At more advanced stages of tumor evolution, we observed significant activation of canonical oncogenic pathways including Wnt, NF-κB, and PI3K, which validates the reliability of our single-cell TEM. Despite existing targeted therapies against these pathways, their clinical efficacy remains limited due to tumor heterogeneity and acquired drug resistance 31-33. Notably, beyond these canonical oncogenic pathways, we detected a marked increase in metabolic activity, which may suggest the extensive metabolic reprogramming that occurs during tumor evolution (Fig. 2E). To gain further insight into this phenomenon, we conducted a detailed analysis of metabolism-associated pathways using single-cell TEM, which revealed glucose metabolism as the most significantly altered pathway (Fig. 2F-H). Consistently, analysis of the TCGA cohort revealed strong correlations among evolutionary core genes, HCC malignancy characteristics, and glucose metabolism (Fig. S2D-F). To further validate these observations, we used the murine TEM to demonstrate upregulation of glycolysis-related genes (Fig. 2I), accompanied by elevated pyruvic acid and lactate levels in advanced-stage HCC tumors (Fig. 2J and K). Preliminary data indicated that glucose significantly promotes tumor progression. To address ethical considerations, we substantially reduced the number of tumor cells injected subcutaneously in the *in vivo* assays. These assays demonstrated that enhanced flux through glucose metabolic pathways directly drives the aggressive behavior of HCC (Fig. 2L-M). Consistent with this, *in vitro* experiments yielded similar results (Fig. S2G-L). This metabolic shift, defined by an increased reliance on aerobic glycolysis (the Warburg effect), facilitates critical processes such as tumor survival, proliferation and migration 34, 35. These findings underscore that glucose metabolic reprogramming serves as a critical driver of HCC evolution by promoting glycolytic activity.

### Malignant cells suppress CD8^+^ T Cell glucose metabolism to promote immune evasion

**Figure 3:**
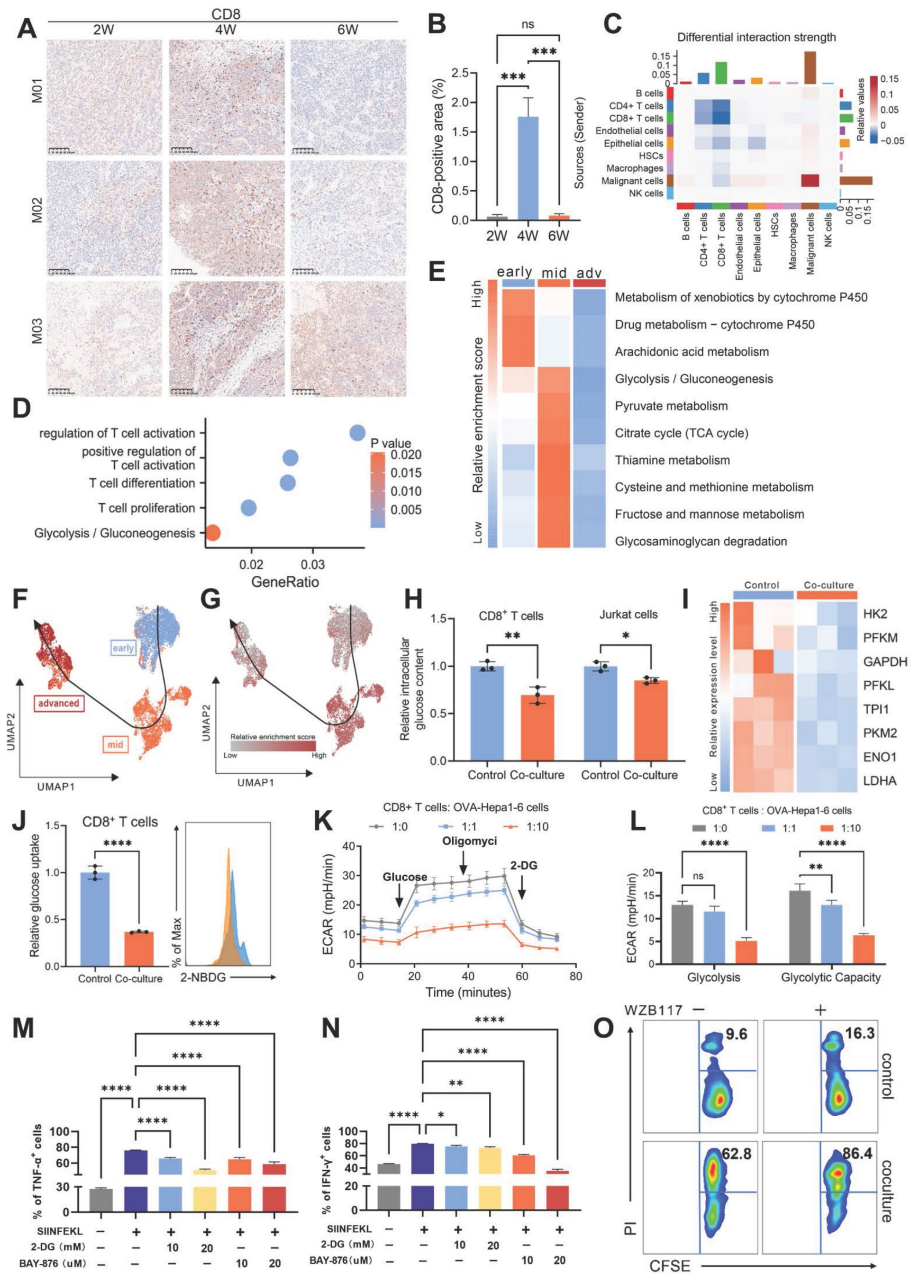
**Malignant cells suppress CD8^+^ T cells glucose metabolism to promote immune evasion. A.** Immunohistochemical staining of CD8 in the tumor samples from murine TEM, with brown signals indicating positive staining. M01-M03 represent three independent biological replicates (n=3). **B.** Quantification of CD8-positive areas was performed in the tumor samples from murine TEM. **C.** The heatmap illustrates the changes in cell-cell interaction strength between advanced and early/mid samples as assessed by CellChat analysis. Cell type annotations are indicated along the axes, with colors representing the relative strength of interactions. **D.** Gene Ontology (GO) enrichment analysis of characteristic genes associated with advanced stage CD8^+^ T cells. **E.** Heatmap depicting GSVA scores of metabolism-related pathways in CD8^+^ T cells across early, mid, and advanced stages of HCC evolution. **F.** UMAP plot showing the reconstructed evolutionary trajectory of CD8^+^ T cells using the Slingshot algorithm. Cells from early, mid, and advanced stages are classified according to the single-cell TEM. **G.** UMAP plot demonstrating GSVA scores for the glycolysis pathway along the Slingshot-inferred trajectory of CD8^+^ T cells. **H.** OT-1 CD8^+^ T cells were co-cultured with OVA-Hepa1-6 cells, while Jurkat cells were co-cultured with Huh7 cells (all at 1:1 ratio) for 48 hours in medium containing 5.5 mM glucose. Subsequently, intracellular glucose levels were measured separately in CD8^+^ T cells (**left**) and Jurkat cells (**right**). **I.** Glycolysis-related gene expression in OT-1 CD8^+^ T cells was compared between control (isolated) and OVA-Hepa1-6 co-cultured groups (1:1 ratio in 5.5 mM glucose medium). All expression levels were normalized to β-actin. **J.** Changes in glucose uptake capacity in OT-1 CD8^+^ T cells following 48 hours of co-culture with OVA-Hepa1-6 cells at a 1:1 ratio in the medium containing 5.5 mM glucose, measured using flow cytometry to detect fluorescence intensity of 2-NBDG. **K and L.** Following co-culture with OVA-Hepa1-6 cells at 1:1 or 1:10 ratios in 5.5 mM glucose medium for 48 hours, CD8^+^ T cells were analyzed for extracellular acidification rate (ECAR) using sequential exposure to 10 mM glucose, 1 μM oligomycin, and 50 mM 2-DG (**K**). Three replicate measurements were performed per condition. Glycolysis was calculated as ECAR increase post-glucose addition, while glycolytic capacity represented the difference between maximal ECAR (post-oligomycin) and ECAR following 2-DG inhibition (**L**). **M and N.** OT-1 CD8^+^ T cells were activated with the OVA-derived peptide SIINFEKL for five days. The culture medium was supplemented with either 10 mM or 20 mM of 2-DG and either 10 µM or 20 µM of BAY-876. Expression levels of TNF-α and IFN-γ were analyzed by flow cytometry, with percentages of TNF-α^+^ (**M**) and IFN-γ^+^ (**N**) cells represented as bar graphs. **O.** The upper panel shows basal death of OVA-Hepa1-6 cells cultured in 5.5 mM glucose medium ±10 µM WZB117 (without CD8^+^ T cells) as control. The lower panel demonstrates cytotoxic activity of activated OT-1 CD8^+^ T cells against OVA-Hepa1-6 cells after 24 hours co-culture in 5.5 mM glucose medium. Data are mean ± SD. *p < 0.05, **p < 0.01, ***p < 0.001, ****p < 0.0001, ns, non-significant by Student's t test (H and J) or by two-way ANOVA (B and L-N).

The TIME is a complex ecosystem where malignant, stromal, and immune cells interact to drive tumor evolution 36. Metabolic reprogramming in malignant cells potentially impairs the metabolism and functionality of surrounding cells 37, 38. To unravel these intricate interdependencies, we employed the single-cell TEM to map the dynamic landscape of the TIME. In the early stage of tumor evolution, there was a pronounced increase in immune cell infiltration, which may represent an initial “activation” phase. However, in advanced stage HCC, we observed a significant reduction in several key immune populations, including B cells, CD4^+^ T cells, CD8^+^ T cells, and macrophages (Fig. 3A-B and Fig. S3A). Analysis of cell-cell interactions demonstrated that inhibitory signaling from malignant cells targeting CD8^+^ T cells was markedly amplified during the advanced stage (Fig. 3C and Fig. S3B). Enrichment analysis further revealed significant impairments in CD8^+^ T cell activation, proliferation, and differentiation, indicating a progressive disruption of their effector functionality (Fig. 3D). Given the well-established critical role of CD8^+^ T cells in mediating cytotoxic responses against tumor cells 39, 40, we propose that this suppression by malignant cells likely plays a pivotal role in facilitating tumor evolution.

In addition to functional impairments, CD8^+^ T cells exhibited significant metabolic changes during tumor evolution, with the most prominent being a marked downregulation of glycolytic activity during the advanced stage (Fig. 3D-E and Fig. S3C). Trajectory analysis also revealed that glycolytic activity, consistent with trends in CD8^+^ T cells' functionality and infiltration levels, initially increased but later declined as the tumor evolved (Fig. 3F-G). Notably, this decline in CD8^+^ T cells glucose metabolism stands in stark contrast to the increased metabolic activity observed in malignant cells during tumor evolution, as previously highlighted.

Based on these observations, we hypothesized that metabolic competition for glucose might occur between malignant cells and CD8^+^ T cells. We established a co-culture system using OVA-specific TCR transgenic OT-1 CD8^+^ T cells and Hepa1-6 cells engineered to express the ovalbumin (OVA) antigen (OVA-Hepa1-6), simulating interactions between malignant cells and CD8^+^ T cells in the TIME. The results revealed that co-culture with malignant cells led to a significant reduction in intracellular glucose levels within CD8^+^ T cells (Fig. 3H), accompanied by marked downregulation of key glycolytic genes (Fig. 3I), indicating impaired glycolytic function. Consistent with these findings, 2-NBDG uptake assays demonstrated a significant decrease in glucose uptake capacity (Fig. 3J), while extracellular acidification rate (ECAR) analysis further confirmed reductions in both basal glycolytic activity and maximum glycolytic capacity (Fig. 3K and L). We also employed activated Jurkat cells as an independent model co-cultured with HCC cells, which exhibited similar metabolic disruptions, further supporting the hypothesis that interactions with malignant cells impair T cell glycolysis (Fig. 3H and Fig. S3D-G).

To assess the impact of impaired glucose metabolism on CD8^+^ T cell functionality, we treated cells with 2-DG, a glucose analog, and BAY-876 41, a glucose transporters (GLUTs) inhibitor. This metabolic inhibition resulted in a significant reduction in the proportion of CD8^+^ T cells expressing key effector molecules, including TNF-α, IFN-γ, GZMB, and perforin (Fig. 3M-N and Fig. S3H-I). GLUT1 serves as the predominant glucose transporter in tumor cells, while CD8^+^ T cells primarily utilize GLUT3 for glucose uptake (Fig. S3J and K) 42. So we employed WZB117 43, a selective GLUT1 inhibitor, to disrupt glucose metabolism in malignant cells. This inhibition significantly increased the sensitivity of tumor cells to the cytotoxic effects of CD8^+^ T cells (Fig. 3O). Collectively, these findings demonstrate that malignant cells actively drive T cell dysfunction by suppressing glycolytic metabolism, thereby facilitating immune evasion and advancing tumor evolution.

### NSUN2-mediated m^5^C modification regulates metabolic reprogramming in a glucose-dependent manner

**Figure 4:**
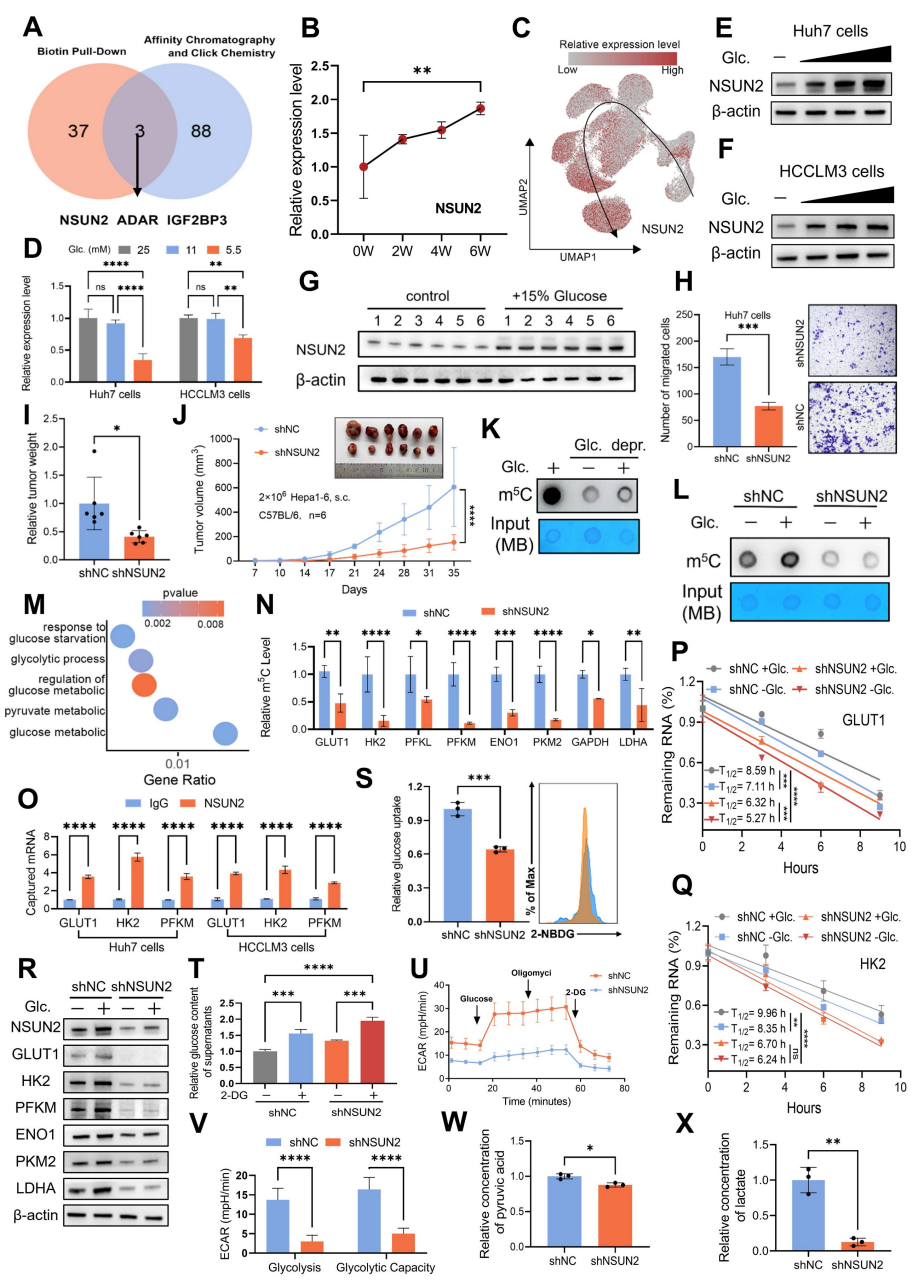
** NSUN2-mediated m^5^C modification regulates metabolic reprogramming in a glucose-dependent manner. A.** Through the intersection of publicly available data from biotin-labeled glucose pull-down, affinity chromatography, and click chemistry analyses, NSUN2, ADAR and IGF2BP3 were identified as candidate proteins that directly interact with glucose. **B.** The expression level of NSUN2 in the murine TEM. **C.** UMAP plot demonstrating the expression level of NSUN2 in the single-cell TEM. **D.** Real-time qPCR analysis of Huh7 and HCCLM3 cells starved with glucose and restoration at indicated concentrations (25, 11, and 5.5 mM) (n = 3 biological replicates). **E and F.** Immunoblotting analysis of Huh7 (**E**) and HCCLM3 (**F**) cells after 6 hours of glucose starvation followed by 2 hours of glucose restoration at indicated concentrations (25, 11, and 5.5 mM). **G.** Immunoblot analysis from subcutaneous tumor models established using Hepa1-6 cells in C57BL/6 mice. The experimental group was provided with drinking water containing 15% glucose, while the control group received regular drinking water (n = 6 animals per group). **H.** Transwell migration assays assessing the migratory capacity of shNC and shNSUN2 Huh7 cells (n = 3 biological replicates). **I and J.** Tumor weight (**I**) and volumes (**J**) were measured in the *in vivo* syngeneic tumorigenesis assay of shNC and shNSUN2 Hepa1-6 cells subcutaneously inoculated in C57BL/6 mice (n = 6 animals/group). **K.** Huh7 cells without or with glucose starvation for 4 hours and restored with glucose (5.5 mM) 2 hours before dot blot assay of m^5^C levels (total RNA) (n = 3 biological replicates). **L.** shNC and shNSUN2 Huh7 cells were glucose starved for 4 hours and restored with glucose (5.5 mM) 2 hours for dot blot assay (n = 3 biological replicates). **M.** GO enrichment analysis of the genes with differential m^5^C methylation levels between shNC and shNSUN2 cells. **N.** RNA-IP using anti-m^5^C antibody, followed by real-time qPCR analysis in shNC and shNSUN2 Huh7 cells with glucose (5.5 mM) (n = 3 biological replicates). **O.** RT-qPCR was applied for detection of endogenous GLUT1, HK2, PFKM mRNA immunoprecipitated with NSUN2 (1:100, 4 °C, overnight). **P and Q.** RNA decay assay in shNC and shNSUN2 Huh7 cells treated with actinomycin D (Act. D, 5 μg/mL), glucose starved and restored with glucose. Real-time qPCR against β-actin was performed to assess the half-life of GLUT1 (**P**) and HK2 (**Q**) mRNA (n = 3 biological replicates). **R.** Immunoblotting analysis of shNC and shNSUN2 Huh7 cells after 6 hours of glucose starvation and subsequent 2 hours of restoration with 5.5 mM glucose. **S.** The glucose uptake capacity in shNC and shNSUN2 Huh7 cells, measured using flow cytometry to detect fluorescence intensity of 2-NBDG (n = 3 biological replicates). **T.** Glucose content in the supernatant of shNC and shNSUN2 Huh7 cells after 48 hours of treatment with or without 5.5 mM 2-DG. **U and V.** Measurements were recorded over time, with exposure to glucose, oligomycin, and 2-DG for ECAR assessment. ECAR in shNC and shNSUN2 Huh7 cells was recorded three times per condition (**U**). Glycolysis (ECAR following glucose addition) and glycolytic capacity (maximal ECAR after subtracting the ECAR following 2-DG exposure) were calculated (**V**). **W and X.** Comparison of the relative pyruvic acid (**W**) and lactate (**X**) production between shNC and shNSUN2 Huh7 cells. Data are mean ± SD. *p < 0.05, **p < 0.01, ***p < 0.001, ****p < 0.0001, ns, non-significant by Student's t test (I-J, N-O, S and V-X) or by two-way ANOVA (B, D, P-Q and T).

Nutrients within the TIME are known as direct substrates in metabolic pathways 44. However, emerging evidence suggests that some nutrients may also function as independent signaling molecules, modulating cellular signaling through mechanisms distinct from conventional metabolism. For example, leucine has been demonstrated to directly activate mTORC1 signaling by binding to its specific receptor protein, SAR1B 45. Building on these findings, we propose whether glucose acquired by malignant cells might similarly serve as a signaling molecule, potentially contributing to HCC evolution. To support this hypothesis, we analyzed findings from previous studies. Weili Miao *et al.* employed affinity purification and chemical crosslinking strategies to identify 91 glucose-binding proteins 21. Tingjin Chen *et al.* identified 40 potential glucose-interacting proteins using a biotin-based pull-down approach followed by mass spectrometry analysis 46. Notably, only three proteins were consistently detected in both studies: NSUN2, ADAR, and IGF2BP3 (Fig. 4A). Among these, NSUN2 emerges as the only transcript consistently upregulated in both murine and single-cell TEMs (Fig. 4B-C and Fig. S4A-D). Moreover, NSUN2 exhibited significant expression changes in response to glucose deprivation and restoration at both the mRNA and protein levels (Fig. 4D-F and Fig. S4E). Similarly, *in vivo*, tumors from mice supplied with glucose-supplemented drinking water exhibited increased NSUN2 expression (Fig. 4G), supporting its role as a glucose-responsive regulator in HCC. Functional assays further confirmed the oncogenic properties of NSUN2, demonstrating its ability to promote tumorigenesis, accelerate cellular proliferation, and enhance migratory capacity (Fig. 4H-J and Fig. S4F-H). Collectively, these findings identify NSUN2 as a potential glucose sensor, highlighting its critical role in driving tumor evolution.

As NSUN2 is the key enzyme mediating global m^5^C RNA methylation in various cancer cell lines 47, 48, we further found that both glucose deprivation and NSUN2 deficiency resulted in a marked decrease in global m^5^C RNA methylation levels (Fig. 4K-L and Fig. S4I-K). Analysis of methylated RNA immunoprecipitation sequencing (MeRIP-seq) data from shNC and shNSUN2 cells revealed that NSUN2-mediated mRNA methylation regulates key pathways associated with glucose metabolism, such as pyruvate metabolism and cellular responses to glucose starvation (Fig. 4M and Fig. S4L; Supplementary Table 2). Moreover, in the TCGA-LIHC cohort, we observed a significant positive correlation between m^5^C modification abundance and NSUN2 RNA expression levels, both of which were closely associated with glucose metabolism (Fig. S4M and N). Supporting these findings, MeRIP experiment confirmed that NSUN2 facilitates the m^5^C modification of multiple glycolytic enzyme mRNAs (Fig. 4N). Given NSUN2's binding to target mRNAs (GLUT1, HK2, and PFKM; Fig. 4O) and the extensively characterized role of m^5^C modifications in mRNA stabilization 49, 50, our results demonstrate that both glucose deprivation and NSUN2 depletion markedly decrease the stability of these key glycolytic enzyme transcripts (Fig. 4P-Q and Fig. S4O). Notably, malignant cells possess an inherent advantage in the competition for glucose, as NSUN2-mediated m^5^C modifications of GLUT1 enhance this advantage by facilitating efficient glucose acquisition, thereby establishing a positive feedback loop that further enables tumor cells to outcompete surrounding cells for available resources. The stability of GLUT1, HK2, and PFKM mRNAs likely drives the widespread alterations in protein expression across the glycolytic pathway (Fig. 4R and Fig. S4P). Meanwhile, NSUN2 depletion led to reduced glucose uptake and consumption (Fig. 4S-T and Fig. S4Q-R), accompanied by a diminished extracellular acidification rate (ECAR) (Fig. 4U-V and Fig. S4S-T) and decreased production of pyruvate and lactate (Fig. 4W-X and Fig. S4U-V). While ATP content alone may not fully capture the complexity of metabolic reprogramming, its correlation with NSUN2 expression reveals corresponding alterations in energy metabolism (Fig. S4W). Collectively, these findings suggest that NSUN2-mediated m^5^C modification plays a pivotal role in regulating metabolic reprogramming.

### The glucose-competition/NSUN2 axis drives tumor evolution and CD8^+^ T cell dysfunction

**Figure 5:**
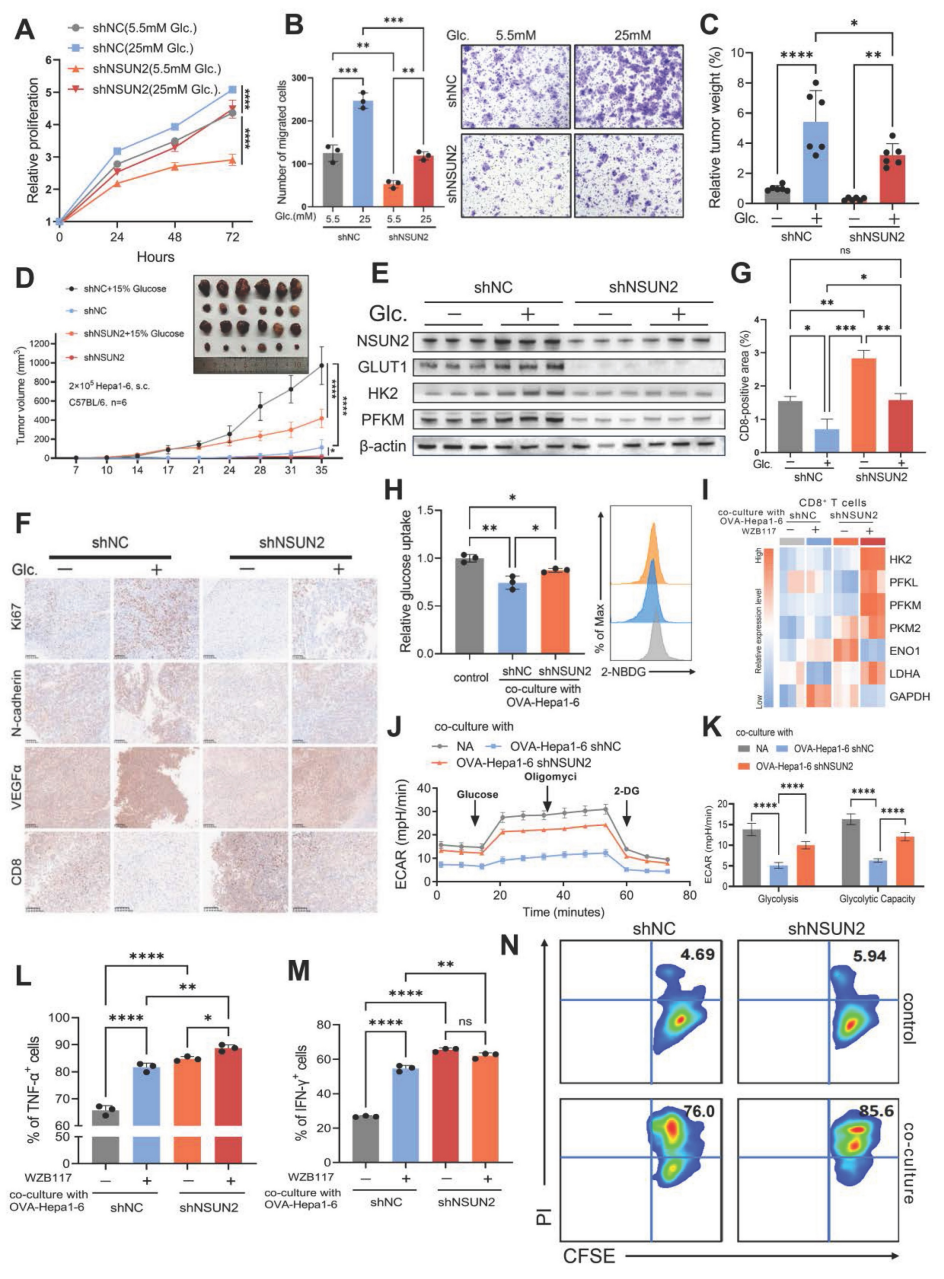
** The glucose-competition/NSUN2 axis drives tumor evolution and CD8^+^ T cells dysfunction. A.** Proliferation of shNC and shNSUN2 Huh7 cells, evaluated by CCK-8 (Cell Counting Kit-8) assays (n = 3 biological replicates). Cells were cultured in media containing 5.5 mM or 25 mM glucose. **B.** Transwell migration assays assessing the migratory capacity of shNC and shNSUN2 Huh7 cells (n = 3 biological replicates). Cells were cultured in media containing 5.5 mM or 25 mM glucose. **C-E.** Tumor weight (**C**) and tumor volumes (**D**) were measured were measured in the *in vivo* syngeneic tumorigenesis assay of shNC and shNSUN2 Hepa1-6 cells subcutaneously inoculated in C57BL/6 mice (n = 6 animals/group). Immunoblot analysis (**E**) of key glycolysis proteins in harvested tumor tissues. Mice in the glucose-supplemented group received drinking water containing 15% glucose, while the control group was provided with regular drinking water. **F.** Immunohistochemical staining of Ki67, N-cadherin, VEGFα and CD8 was performed on the *in vivo* syngeneic tumorigenesis assay of shNC and shNSUN2 Hepa1-6 cells subcutaneously inoculated in C57BL/6 mice. Mice in the glucose-supplemented group received drinking water containing 15% glucose, while the control group was provided with regular drinking water. Brown signals indicate positive staining for the respective markers. **G.** Quantification of CD8-positive areas in the tumor samples as described in (**F**). **H.** Glucose uptake in OT-1 CD8^+^ T cells was measured by 2-NBDG fluorescence (flow cytometry) after 48 hours co-culture with shNC or shNSUN2 OVA-Hepa1-6 cells (1:1 ratio) in 5.5 mM glucose medium. **I.** Glycolysis-related gene expression (normalized to β-actin) in OT-1 CD8^+^ T cells following 48 hours co-culture with shNC/shNSUN2 OVA-Hepa1-6 cells (1:1 ratio) in 5.5 mM glucose medium ±10 µM WZB117. **J and K.** ECAR analysis of OT-1 CD8^+^ T cells after 48h co-culture with shNC/shNSUN2 OVA-Hepa1-6 (1:1 ratio) in 5.5 mM glucose medium. Measurements were recorded over time, with exposure to glucose, oligomycin, and 2-DG for ECAR assessment. ECAR was recorded three times per condition (**J**). Glycolysis (ECAR following glucose addition) and glycolytic capacity (maximal ECAR after subtracting the ECAR following 2-DG exposure) were calculated (**K**). **L and M.** OVA-specific TCR transgenic OT-1 CD8^+^ T cells were activated with the OVA-derived peptide SIINFEKL for five days. Subsequently, these activated OT-1 CD8^+^ T cells cells were co-cultured with shNC or shNSUN2 OVA-Hepa1-6 cells at ratios of 1:1 for 48 hours in 5.5 mM glucose medium, in the presence or absence of 10 µM WZB117. The expression levels of TNF-α and IFN-γ in OT-1 CD8^+^ T cells were analyzed by flow cytometry. Percentages of TNF-α^+^ (**L**) and IFN-γ^+^ cells (**M**) are shown as bar graphs. **N.** The upper panel illustrates the natural cell death of shNC and shNSUN2 OVA-Hepa1-6 cells cultured in 5.5 mM glucose medium in the absence of co-culture with OT-1 CD8^+^ T cells, serving as a control for baseline cell death. The lower panel demonstrates the cytotoxic activity of activated OT-1 CD8^+^ T cells toward shNC and shNSUN2 OVA-Hepa1-6 cells following 24 hours of co-culture in 5.5 mM glucose medium. Data are mean ± SD. *p < 0.05, **p < 0.01, ***p < 0.001, ****p < 0.0001, ns, non-significant by two-way ANOVA (A-D, G-H and K-M).

Building on the glucose-competition/NSUN2 axis's role in enhancing the nutrient acquisition advantage of tumor cells, we explored whether this mechanism could influence HCC evolutionary fate and contribute to CD8^+^ T cell functional activity within the TIME. *In vitro,* NSUN2 deficiency significantly impaired the proliferation, survival, and invasiveness of cancer cells, effects that were fully reversed by exogenous glucose supplementation (Fig. 5A-B and Fig. S5A-C). Similarly, *in vivo* subcutaneous tumor formation assays revealed concordant results (Fig. 5C and D). Notably, the regulatory effect of glucose on NSUN2 expression and its downstream modulation of glycolytic enzymes, initially observed *in vitro*, was further validated in tumor tissues (Fig. 5E and Fig. S5D). Furthermore, both glucose deprivation and NSUN2 silencing significantly downregulated the expression of malignancy-associated markers (Fig. 5F and Fig. S5E-G). These data highlight the critical function of the glucose-competition/NSUN2 axis in driving HCC evolutionary trajectory.

And we found that enhancing glucose availability or overexpressing NSUN2 significantly diminished CD8^+^ T cell infiltration (Fig. 5F and G). Based on these findings, we have proposed that tumor cells could boost their glucose metabolism activity via the glucose-competition/NSUN2 axis, consequently hindering the metabolism and anti-tumor capabilities of CD8^+^ T cells. Then we co-cultured CD8^+^ T cells with malignant cells and found that restricting glucose uptake by malignant cells or silencing NSUN2 effectively alleviated tumor-driven metabolic suppression in CD8^+^ T cells (Fig. 5H and Fig. S5H). These interventions notably upregulated the expression of key glycolysis-related genes in CD8^+^ T cells (Fig. 5I and Fig. S5I) and significantly increased the ECAR (Fig. 5J-K and Fig. S5J-K), reflecting the restoration of glycolytic activity. As anticipated, metabolically suppressed or NSUN2-deficient tumor cells exhibited a markedly diminished capacity to inhibit the antitumor functions of CD8^+^ T cells compared to control tumor cells. This was demonstrated by a pronounced increase in the secretion of pro-inflammatory cytokines (Fig. 5L-M and Fig. S5L-M), alongside significantly enhanced cytotoxic activity of CD8^+^ T cells (Fig. 5N). Overall, these findings reveal the glucose-competition/NSUN2 axis as a pivotal driver of tumor evolution and immune evasion, primarily through disrupting CD8^+^ T cell metabolism and suppressing their antitumor activity within the TIME.

### Immunometabolic therapy overcomes HCC resistance to immune checkpoint inhibitors

**Figure 6:**
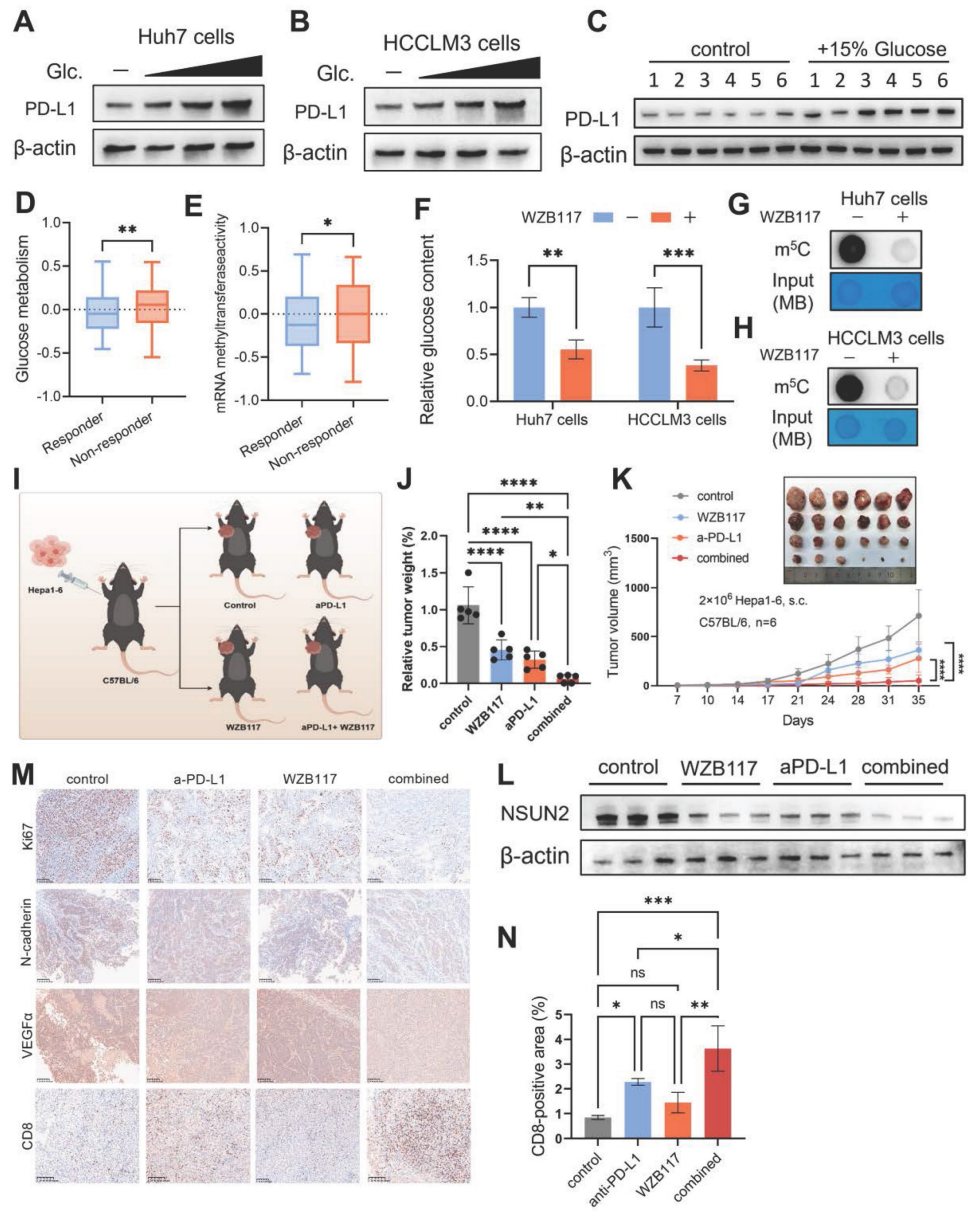
** Immunometabolic therapy overcomes HCC resistance to immune checkpoint inhibitors. A and B.** Immunoblotting analysis of Huh7 (**A**) and HCCLM3 (**B**) cells starved with glucose and restored with indicated concentrations of glucose (25 ,11 and 5.5 mM) (n = 3 biological replicates). **C.** Immunoblot analysis from subcutaneous tumor models established using Hepa1-6 cells in C57BL/6 mice. The experimental group was provided with drinking water containing 15% glucose, while the control group received regular drinking water (n = 6 animals per group). **D and E.** Comparison of mRNA methyltransferase activity (**D**) and glucose metabolism activity (**E**) between immune therapy responders and non-responders, as predicted by the TIDE algorithm in the TCGA-LIHC cohort. **F.** Intracellular glucose levels in Huh7 and HCCLM3 cells with or without 10 μM WZB117 treatment for 8 hours. **G and H.** m^5^C levels (total RNA) in Huh7 (**G**) and HCCLM3 (**H**) cells treated with or without 10 μM WZB117 for 8 hours, as measured by dot blot assay (n = 3 biological replicates). **I.** Schematic diagram illustrating the combined therapy regimen of WZB117 and anti-PD-L1 in the *in vivo* syngeneic tumorigenesis assay of Hepa1-6 cells subcutaneously inoculated in C57BL/6 mice (n = 6 animals per group). **J and K.** Tumor weight (**J**) and tumor volumes (**K**) were measured on the *in vivo* syngeneic tumorigenesis assay of Hepa1-6 cells subcutaneously inoculated in C57BL/6 mice (n = 6 animals per group). The groups included the control group, WZB117 monotherapy, anti-PD-L1 monotherapy, and the combined treatment group. **L.** Immunoblotting analysis was performed on the *in vivo* syngeneic tumorigenesis assay of Hepa1-6 cells subcutaneously inoculated in C57BL/6 mice. The experimental groups included the control group, WZB117 monotherapy, anti-PD-L1 monotherapy, and the combined treatment group. **M.** Immunohistochemical staining for Ki67, N-cadherin, VEGFα, and CD8 was performed on the *in vivo* syngeneic tumorigenesis assay of Hepa1-6 cells subcutaneously inoculated in C57BL/6 mice. The groups included the control group, WZB117 monotherapy, anti-PD-L1 monotherapy, and the combined treatment group. Brown staining indicates positive expression of the respective markers. **N.** Quantification of CD8-positive areas in the tumor samples as described in (**M**). Data are mean ± SD. *p < 0.05, **p < 0.01, ***p < 0.001, ****p < 0.0001, ns, non-significant by Student's t test (D-F) or by two-way ANOVA (J-K and N).

Immune checkpoint inhibitors (ICIs) have revolutionized the treatment landscape for HCC by harnessing the immune system—particularly CD8^+^ T cells, which are pivotal in recognizing and destroying cancer cells through their cytotoxic activity 51, 52. However, despite the promise of ICIs, many HCC patients exhibit primary or acquired resistance, significantly limiting their clinical efficacy 53. Consistent with previous studies 54, 55, our findings show that glucose promotes PD-L1 expression in HCC cells (Fig. 6A and B) as well as in tumor tissues of mice provided with glucose-supplemented drinking water (Fig. 6C). Furthermore, predictions generated by the TIDE (Tumor Immune Dysfunction and Exclusion) algorithm 56 suggest that mRNA methyltransferase activity and glucose metabolism play a pivotal role in shaping the immunotherapy response in HCC patients (Figures 6D and E). Inhibition of glucose transport by WZB117 significantly reduces global RNA m^5^C levels, highlighting a direct regulatory link between glucose metabolism and RNA methylation (Fig. 6F-H). These observations prompted us to explore whether targeting the glucose-competition/NSUN2 axis could overcome resistance to ICIs and improve therapeutic efficacy (Fig. 6I).

The combination of WZB117 and anti-PD-L1 treatments exhibited a significant inhibitory effect on tumor growth in subcutaneous tumor-bearing mice models (Fig. 6J and K). And this dual treatment notably suppressed NSUN2 expression (Fig. 6L), suggesting that such an immunometabolic strategy could potentially alter the evolutionary trajectory of HCC. The marked reduction in malignant tumor characteristics observed with this approach provides strong support for this hypothesis (Fig. 6M). Moreover, this treatment strategy significantly increased the infiltration of CD8^+^ T cells into the TIME (Fig. 6M and N). These findings suggest that the combined WZB117 and anti-PD-L1 therapy effectively targets the glucose-competition/NSUN2 axis in HCC, enhancing CD8^+^ T cell anti-tumor activity while partially regulating HCC evolution through NSUN2-m^5^C modification. By simultaneously targeting metabolic pathways and immune resistance, this combinatorial approach provides new insights into potential therapeutic interventions for HCC.

## Discussion

Our research established a murine TEM and identified genes with significant alterations during tumor evolution through a comprehensive approach, integrating differential expression analysis, WGCNA, machine learning algorithms and Mfuzz clustering. To further refine our findings, snRNA-seq data from resected tumor tissues of 10 HCC patients, supplemented by 11 samples from an external dataset, were utilized to construct a single-cell TEM. This model enables an in-depth exploration of cellular interactions and signaling pathways within the TIME, providing valuable insights into the dynamic processes driving tumor evolution.

Through a comprehensive analysis of the TIME using our model, we identified metabolic reprogramming as the most prominent alteration driving tumor evolution, characterized by the opposing shifts in glucose metabolism observed between tumor cells and CD8^+^ T cells. Tumor cells display a pronounced upregulation of glucose metabolism, which drives their growth and survival, whereas CD8^+^ T cells experience a significant downregulation, leading to a weakened anti-tumor response. This metabolic divergence suggests that tumor cells, by imposing metabolic restrictions on CD8^+^ T cells, outcompete them for nutrients within the TIME, thereby driving tumor evolution and fostering an immunosuppressive microenvironment.

Advanced-stage HCC exhibits significantly enhanced glycolytic activity, also known as the Warburg effect, which is strongly associated with poor prognosis 57, 58. While the mechanisms linking the Warburg effect to tumor evolution remain poorly understood, recent studies have identified a non-metabolic role of glucose. Specifically, glucose can directly interact with certain proteins, termed glucose sensors, to regulate various cellular processes independently of classical metabolic pathways 21, 46. Among these proteins, the expression level of NSUN2 is dynamically upregulated during evolution, with its expression regulated by glucose in a dose-dependent manner. Furthermore, we demonstrate that NSUN2 plays a pivotal role in driving the malignant characteristics of HCC cells. Consistently,* in vivo*, NSUN2 overexpression or additional glucose supplementation significantly enhances tumor growth. These findings highlight the critical role of the glucose-competition/NSUN2 axis in tumor evolution.

NSUN2 serves as the primary writer for m^5^C RNA methylation, and its aberrant expression or mutation is closely associated with tumor initiation and progression 59. Through MeRIP-seq analysis and MeRIP experiments, we found that NSUN2 deficiency significantly reduces m^5^C modification levels in the mRNAs encoding key glucose metabolism enzymes, including GLUT1, HK2, and PFKM. Notably, this process involves a positive feedback loop initiated by glucose uptake in tumor cells, which upregulates NSUN2 expression. In turn, NSUN2 enhances the stability of GLUT1 mRNA, the primary glucose transporter in tumor cells, thereby promoting increased glucose uptake. Further, our study demonstrates that targeting the glucose-competition/NSUN2 axis in malignant cells not only impacts their evolutionary trajectory but also alleviates their metabolic suppression of CD8^+^ T cells. This intervention enhances CD8^+^ T cell infiltration and anti-tumor activity within the TIME.

The widespread adoption of immune checkpoint inhibitors (ICIs) has ushered in a new era of cancer therapy 60. However, most patients still fail to benefit from immunotherapy, likely due to the immunosuppressive characteristics of the TIME 61, 62. Recent advances in understanding tumor metabolic reprogramming and the intricate metabolic interplay between tumor and immune cells have provided new strategies for targeting tumor immunometabolism, offering opportunities to enhance antitumor immune responses and improve therapeutic outcomes 63. Our research provides a novel treatment strategy utilizing WZB117 to disrupt the glucose-competition/NSUN2 axis in malignant cells. This method tackles the challenge of resistance to anti-PD-L1 immunotherapy by hindering metabolic reprogramming in malignant cells and activating CD8^+^ T cells. Besides that, we discovered that the combined application of these treatments can significantly reduce the expression of NSUN2, ultimately partially preventing the uncontrolled evolution of HCC, suggesting a treatment strategy with promising clinical application potential.

In summary, using murine and single-cell TEMs, we reveal that the glucose-competition/NSUN2 axis drives metabolic reprogramming in malignant cells through an m^5^C modification-dependent manner, serving as a key driver of HCC evolution. Furthermore, WZB117 demonstrates potential as a sensitizer for ICI therapy by disrupting the ongoing evolution of HCC. Our findings provide new insights into metabolic reprogramming during tumor evolution and offer potential strategies to overcome immunotherapy resistance.

While our study established the critical role of the glucose-competition/NSUN2 axis in tumor evolution from phenotypic, mechanistic, and therapeutic perspectives, a more detailed understanding of NSUN2's downstream targets and the functional outcomes of m^5^C deposition is necessary to further refine this regulatory framework. Although our findings primarily highlight glucose metabolism, m^5^C modifications may also affect other metabolic pathways such as lipid and amino acid metabolism, which deserve further investigation to clarify their contribution to tumor evolution.

## Conclusion

This study constructed murine and single-cell TEMs to systematically investigate intratumoral heterogeneity and the TIME from a dynamic evolutionary perspective. The results identify NSUN2 as a critical glucose sensor that drives tumor-immune glucose metabolic reprogramming through m^5^C modification. Beyond elucidating the role of the metabolic-immune crosstalk axis in tumor evolution, this work also proposed a novel combinatorial therapeutic strategy, providing new directions for anti-tumor treatment.

## Supplementary Material

Supplementary figures, tables, and materials.

## Abbreviations

HCC: Hepatocellular carcinoma
TIME: Tumor immune microenvironment
TEM: Tumor evolution model
ECAR: Extracellular acidification rates
SAM: S-adenosylmethionine
TAMs: Tumor-associated macrophages
DEGs: Differentially expressed genes
WGCNA: Weighted gene co-expression network analysis
GSVA: Gene set variation analysis
GO: Gene Ontology
m^5^C: 5-methylcytosine
SVM-RFE: Support Vector Machine-Recursive Feature Elimination
Mfuzz: Fuzzy c-means clustering analysis
scRNA-seq: Single-cell RNA sequencing
UMAP: Uniform Manifold Approximation and Projection
MeRIP: Methylated RNA immunoprecipitation
ICIs: Immune checkpoint inhibitors
TIDE: Tumor Immune Dysfunction and Exclusion
