# Supplementary material for: The glucose sensor NSUN2-m5C modification regulates tumor-immune glucose metabolism reprogramming to drive hepatocellular carcinoma evolution
Source: Int J Biol Sci. 2025 Jul 11;21(10):4529–48. doi: 10.7150/ijbs.115610 (PMC12320233; doi:10.7150/ijbs.115610)
Supplement: Supplementary file 1 — Supplementary figures, tables, and materials. [file ijbsv21p4529s1.pdf]

Supplementary Data and Materials

The glucose sensor NSUN2-m^{5}C modification regulates tumor-immune glucose metabolism reprogramming to drive hepatocellular carcinoma evolution

Catalogue

Supplementary Figure **2**

Fig. S1 2

Fig. S2 4

Fig. S3 7

Fig. S4 10

Fig. S5 13

Supplementary Table 16

Supplementary Table 1 16

Supplementary Table 2 17

Supplementary Materials **18**

Fig. S1

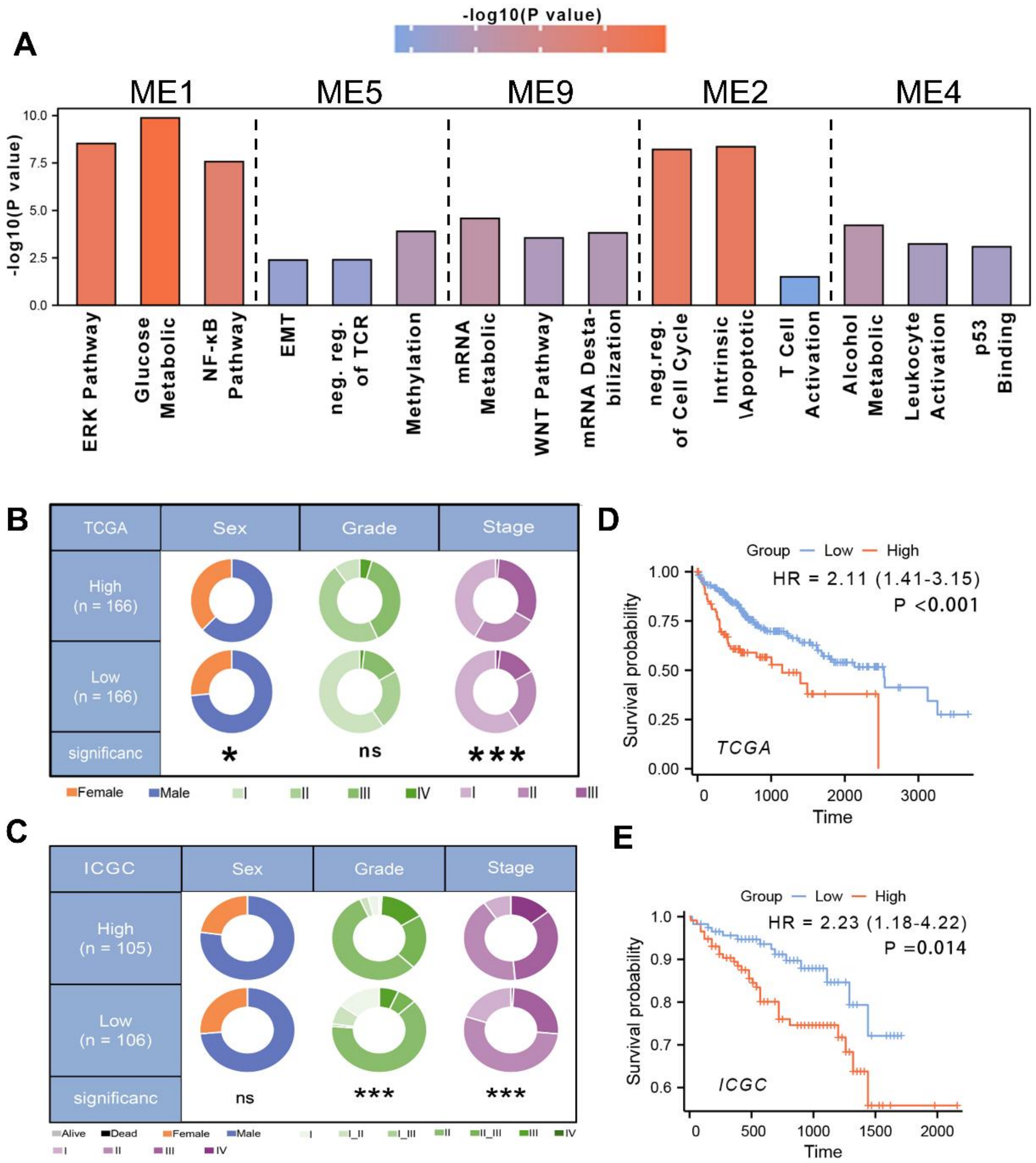
A
-log10(P value)
ME1
ME5
ME9
ME2
ME4
ERK Pathway
Glucose Metabolic
NF-κB Pathway
EMT
neg. reg. of TCR
Methylation
mRNA Metabolic
WNT Pathway
mRNA Destabilization
neg.reg. of Cell Cycle
Intrinsic \Apoptotic
T Cell Activation
Alcohol Metabolic
Leukocyte Activation
p53 Binding
B
TCGA
Sex
Grade
Stage
High (n = 166)
Low (n = 166)
significanc
*
ns
***
Female
Male
C
ICGC
High (n = 105)
Low (n = 106)
D
Group — Low — High
HR = 2.11 (1.41-3.15)
P <0.001
Survival probability
TCGA
Time
E
HR = 2.23 (1.18-4.22)
P =0.014
ICGC

Fig. S1. Identification of evolutionary core genes in the murine tumor evolution model (TEM), related to Fig. 1.

A. Gene Ontology (GO) enrichment analysis of WGCNA modules showing stepwise expression changes in the murine TEM: upregulated modules ME1, ME5, and ME9, and downregulated modules ME2 and ME4.

B and C. Pie charts visually representing the associations between clinicopathological factors and the expression of evolutionary core genes (clusters C1 and C4) in the TCGA-LIHC (**B**) and ICGC (**C**) cohort. *$p < 0.05$, **$p < 0.01$, ***$p < 0.001$, ****$p < 0.0001$, ns, non-significant by Chi-square test.

D and E. Kaplan-Meier survival curves comparing high- and low-expression groups of evolutionary core genes (clusters C1 and C4), with hazard ratios (HR) and p-values calculated using Cox proportional hazards regression analysis in the TCGA-LIHC (**D**) and ICGC (**E**) cohorts.

Fig. S2

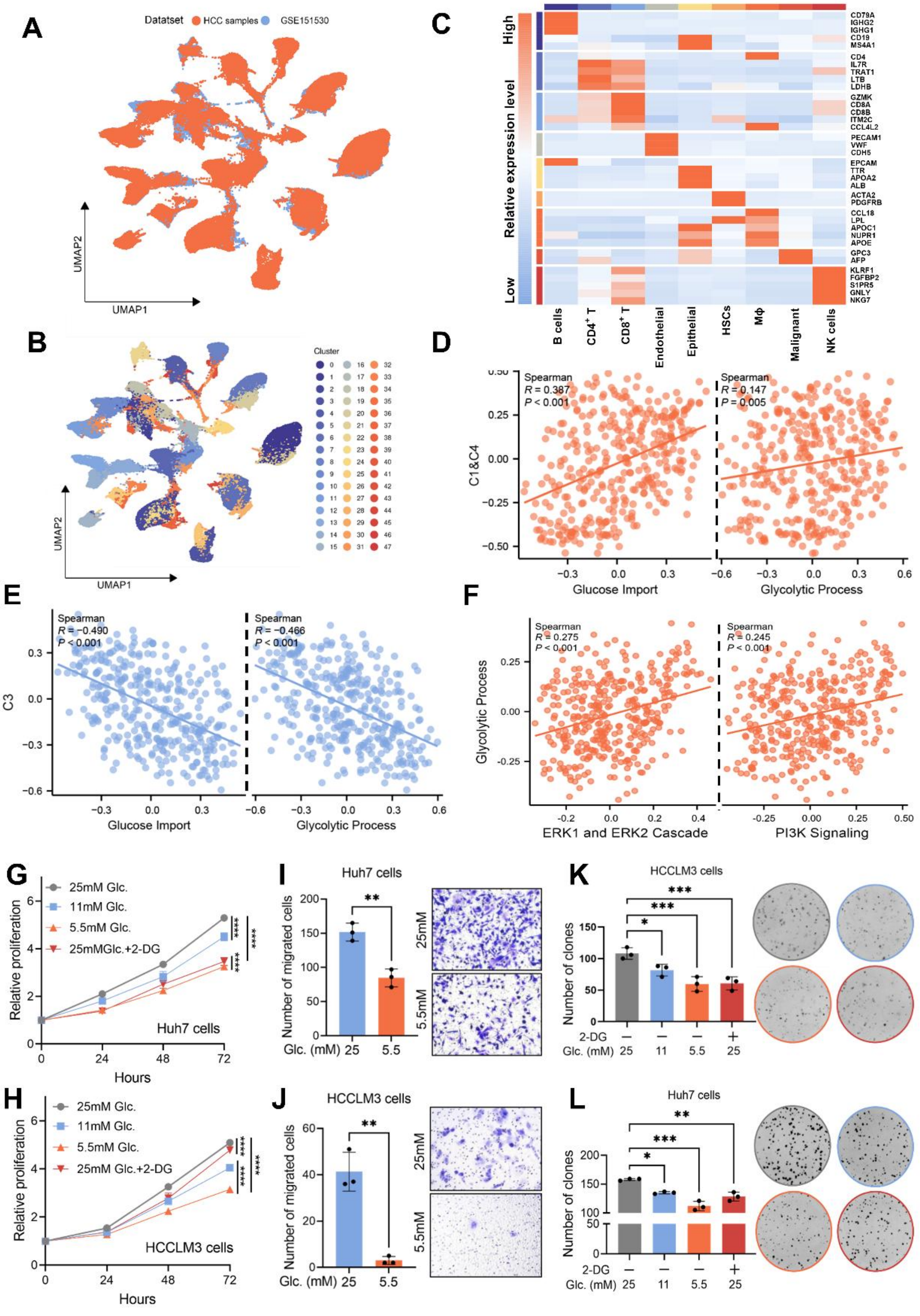

A
Datatset HCC samples GSE151530
UMAP2
UMAP1
B
Cluster
UMAP2
UMAP1
C
High
Relative expression level
Low
CD79A
IGHG2
IGHG1
CD19
MS4A1
CD4
IL7R
TRAT1
LTB
LDHB
GZMK
CD8A
CD8B
ITM2C
CCL4L2
PECAM1
VWF
CDH5
EPCAM
TTR
APOA2
ALB
ACTA2
PDGFRB
CCL18
LPL
APOC1
NUPR1
APOE
GPC3
AFP
KLRF1
FGFBP2
S1PR5
GNLY
NKG7
B cells
CD4+ T
CD8+ T
Endothelial
Epithelial
HSCs
MΦ
Malignant
NK cells
D
Spearman
R = 0.387
P < 0.001
Spearman
R = 0.147
P = 0.005
C1&C4
Glucose Import
Glycolytic Process
E
Spearman
R = −0.490
P < 0.001
Spearman
R = −0.466
P < 0.001
C3
Glucose Import
Glycolytic Process
F
Spearman
R = 0.275
P < 0.001
Spearman
R = 0.245
P < 0.001
Glycolytic Process
ERK1 and ERK2 Cascade
PI3K Signaling
G
25mM Glc.
11mM Glc.
5.5mM Glc.
25mMGlc.+2-DG
Relative proliferation
Huh7 cells
Hours
H
25mM Glc.
11mM Glc.
5.5mM Glc.
25mM Glc.+2-DG
Relative proliferation
HCCLM3 cells
Hours
I
Huh7 cells
Number of migrated cells
Glc. (mM) 25 5.5
25mM
5.5mM
J
HCCLM3 cells
Number of migrated cells
Glc. (mM) 25 5.5
25mM
5.5mM
K
HCCLM3 cells
Number of clones
2-DG − − − +
Glc. (mM) 25 11 5.5 25
L
Huh7 cells
Number of clones
2-DG − − − +
Glc. (mM) 25 11 5.5 25

Fig. S2. Glucose metabolic reprogramming is a crucial event during HCC evolution, related to Fig. 2.

A. UMAP plot showing the batch-correction results of single-nucleus RNA sequencing (snRNA-seq) data from 10 HCC patient samples integrated with external GEO dataset GSE151530 (n = 11) using the LIGER algorithm.

B. UMAP plot displaying the results of dimensionality reduction and unsupervised clustering of single-cell data.

C. Heatmap showing the scaled average expression levels of characteristic marker genes for each cell type.

D and E. Correlation between GSVA enrichment scores for glucose import and glycolytic process pathways with temporal Mfuzz clusters in the TCGA-LIHC cohort: clusters C1 and C4 (**D**), and cluster C3 (**E**).

F. Correlation between GSVA enrichment scores for tumor malignancy-associated pathways, including the ERK1/2 cascade and PI3K signaling pathway, with glycolytic process GSVA enrichment scores in the TCGA-LIHC cohort.

G and H. Proliferation of Huh7 cells (**G**) and HCCLM3 cells (**H**) treated with 25 mM, 11 mM or 5.5 mM glucose, or 5.5 mM 2-DG (2-deoxy-D-glucose), measured by CCK-8 assay (n = 3 biological replicates).

I and J. Transwell migration assays evaluating the migratory capacity of Huh7 cells (**I**) and HCCLM3 cells (**J**) treated with 25 mM glucose or 5.5 mM glucose (n = 3 biological replicates).

K and L. Colony formation assays of Huh7 cells (**K**) and HCCLM3 cells (**L**) treated with 25

mM glucose, 11 mM glucose, 5.5 mM glucose, or 5.5 mM 2-DG (n = 3 biological replicates).

Data are mean ± SD. *p < 0.05, **p < 0.01, ***p < 0.001, ****p < 0.0001, ns, non-significant Student's t test (I and J) or by two-way ANOVA (G-H and K-L).

Fig. S3

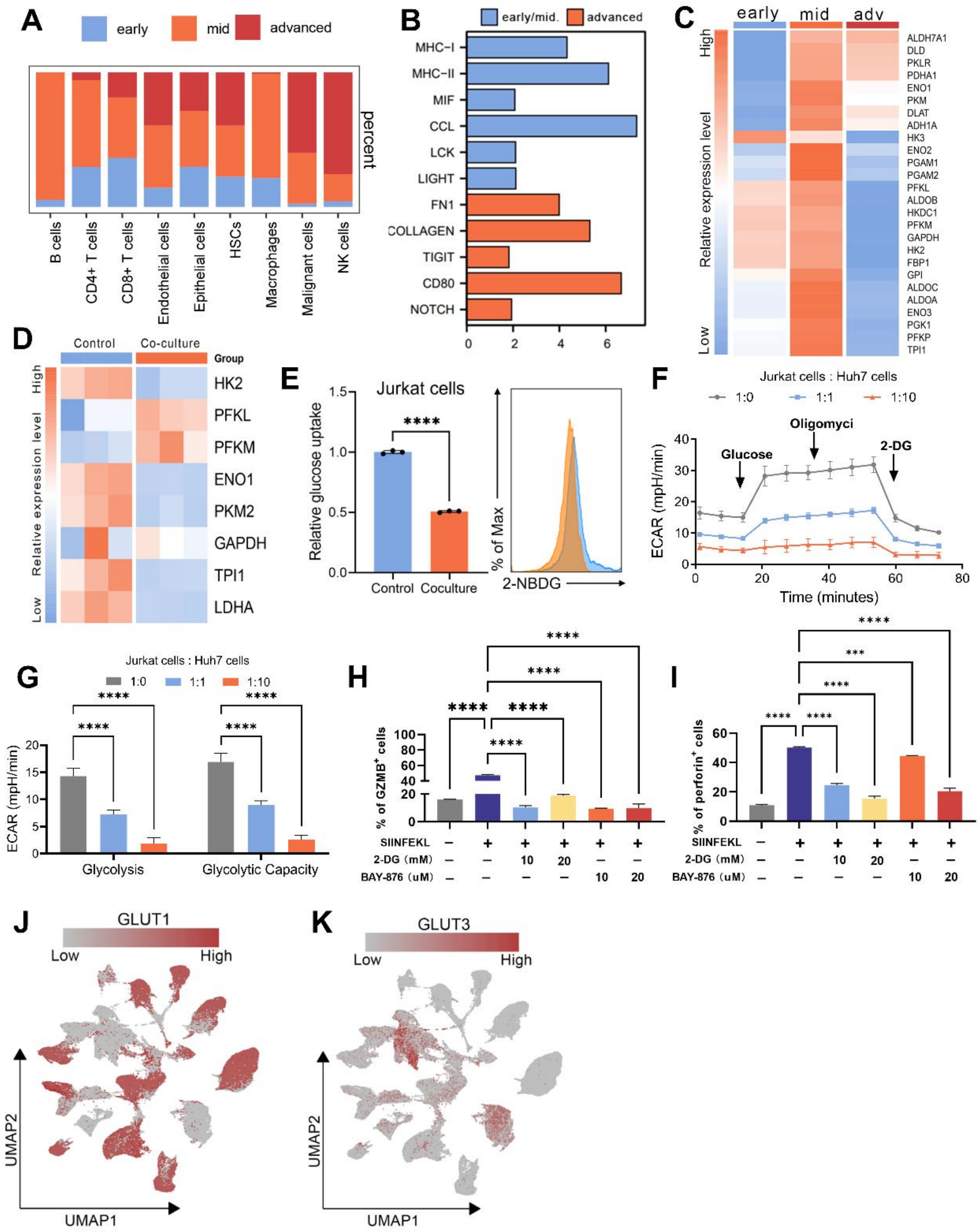

A
early
mid
advanced
percent
B cells
CD4+ T cells
CD8+ T cells
Endothelial cells
Epithelial cells
HSCs
Macrophages
Malignant cells
NK cells
B
early/mid.
advanced
MHC-I
MHC-II
MIF
CCL
LCK
LIGHT
FN1
COLLAGEN
TIGIT
CD80
NOTCH
0
2
4
6
C
early
mid
adv
High
Relative expression level
Low
ALDH7A1
DLD
PKLR
PDHA1
ENO1
PKM
DLAT
ADH1A
HK3
ENO2
PGAM1
PGAM2
PFKL
ALDOB
HKDC1
PFKM
GAPDH
HK2
FBP1
GPI
ALDOC
ALDOA
ENO3
PGK1
PFKP
TPI1
D
Control
Co-culture
Group
HK2
PFKL
PFKM
ENO1
PKM2
GAPDH
TPI1
LDHA
E
Jurkat cells
Relative glucose uptake
Control
Coculture
% of Max
2-NBDG
F
Jurkat cells : Huh7 cells
1:0
1:1
1:10
Glucose
Oligomyci
2-DG
ECAR (mpH/min)
Time (minutes)
G
Jurkat cells : Huh7 cells
Glycolysis
Glycolytic Capacity
H
% of GZMB+ cells
SIINFEKL
2-DG (mM)
BAY-876 (uM)
I
% of perforin+ cells
J
GLUT1
Low
High
UMAP1
UMAP2
K
GLUT3

Fig. S3. Malignant cells suppress $CD8^+$ T Cell glucose metabolism to promote immune evasion, related to Fig. 3.

A. Average proportion of each cell type in samples from early, mid, and advanced stages in single-cell TEM.

B. Significantly upregulated signaling pathways between malignant cells and $CD8^+$ T cells observed in samples from early, mid, and advanced stages of single-cell TEM, as assessed by CellChat analysis.

C. The heatmap illustrates the expression levels of genes involved in the glycolytic pathway of $CD8^+$ T cells across early, mid, and advanced samples of single-cell TEM.

D. Expression levels of glycolysis-related genes in Jurkat cells compared between the control group (isolated) and those co-cultured with Huh7 cells at a 1:1 ratio in 5.5 mM glucose medium, with each gene expression level normalized to β-actin.

E. Changes in glucose uptake capacity in Jurkat cells following 48 hours of co-culture with Huh7 cells at a 1:1 ratio in 5.5 mM glucose medium, measured using flow cytometry to detect fluorescence intensity of 2-NBDG.

F and G. Following a co-culture with Huh7 cells at ratios of 1:1 and 1:10 for 48 hours in 5.5 mM glucose medium, the Jurkat cells were assessed for extracellular acidification rate (ECAR). Measurements were recorded over time, with exposure to glucose, oligomycin, and 2-DG for ECAR assessment. ECAR was recorded three times per condition (**F**). Glycolysis (ECAR following glucose addition) and glycolytic capacity (maximal ECAR after subtracting the ECAR following 2-DG exposure) were calculated (**G**).

H and I. OT-1 $CD8^+$ T cells were activated with the OVA-derived peptide SIINFEKL for five

days. The culture medium was supplemented with either 10 mM or 20 mM of 2-DG and either 10 μM or 20 μM of BAY-876. Expression levels of GZMB and perforin were analyzed by flow cytometry, with percentages of $GZMB^+$ (**H**) and $perforin^+$ (**I**) cells represented as bar graphs.

J and K. UMAP plot showing the expression levels of GLUT1 (**J**) and GLUT3 (**K**) across various cell types within the TIME.

Data are mean ± SD. *p < 0.05, **p < 0.01, ***p < 0.001, ****p < 0.0001, ns, non-significant Student's t test (E) or by two-way ANOVA (G-I).

Fig. S4

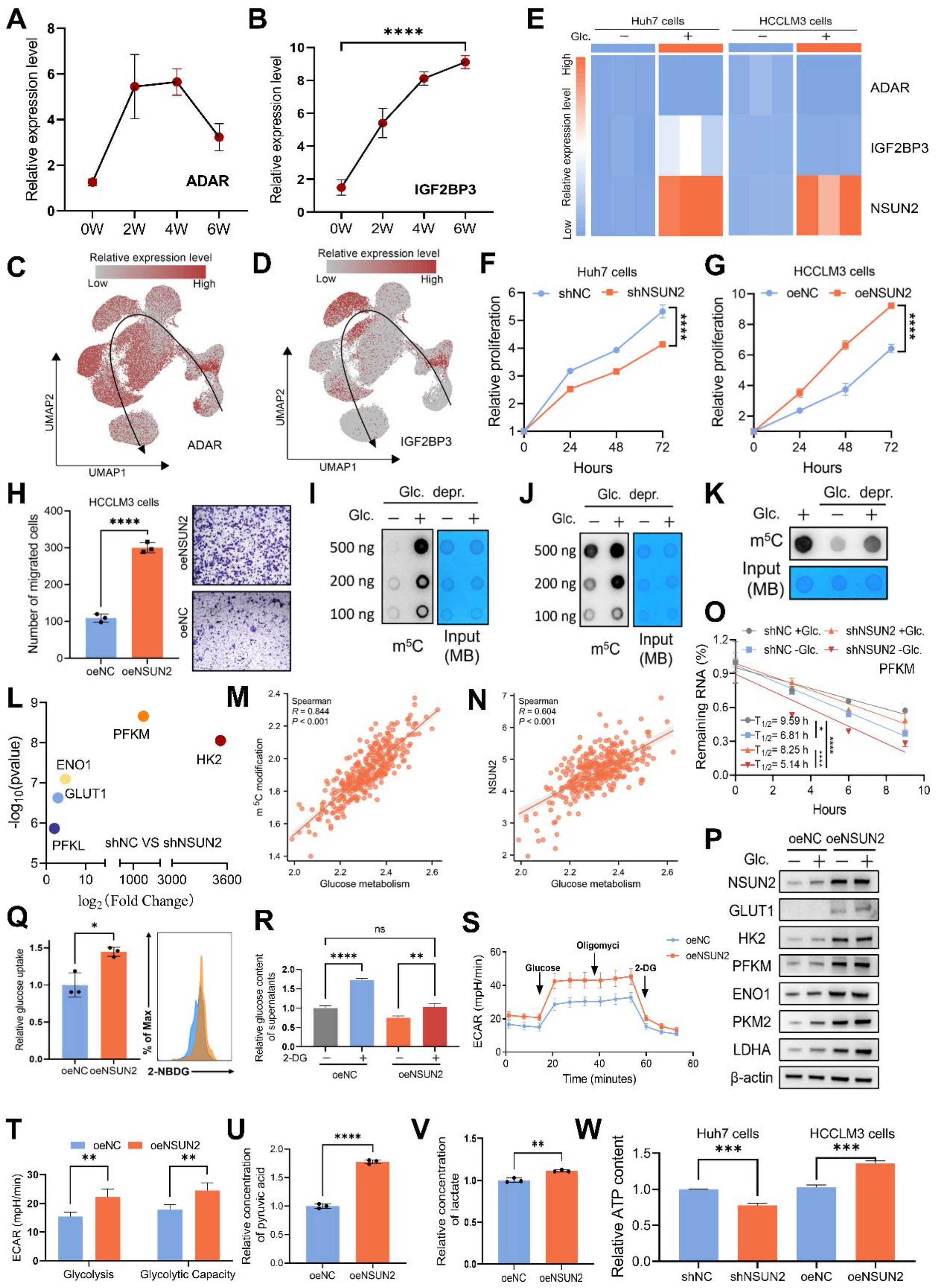
A
Relative expression level
ADAR
0W 2W 4W 6W
B
****
Relative expression level
IGF2BP3
0W 2W 4W 6W
E
Huh7 cells
HCCLM3 cells
Glc. – + – +
High
Relative expression level
Low
ADAR
IGF2BP3
NSUN2
C
Relative expression level
Low High
UMAP2
UMAP1
ADAR
D
Relative expression level
Low High
UMAP2
UMAP1
IGF2BP3
F
Huh7 cells
shNC shNSUN2
Relative proliferation
****
Hours
G
HCCLM3 cells
oeNC oeNSUN2
Relative proliferation
****
Hours
H
HCCLM3 cells
Number of migrated cells
****
oeNC oeNSUN2
oeNSUN2
oeNC
I
Glc. depr.
Glc. – + – +
500 ng
200 ng
100 ng
m5C
Input (MB)
J
Glc. depr.
Glc. – + – +
500 ng
200 ng
100 ng
m5C
Input (MB)
K
Glc. depr.
Glc. + – +
m5C
Input (MB)
O
shNC +Glc. shNSUN2 +Glc.
shNC -Glc. shNSUN2 -Glc.
PFKM
Remaining RNA (%)
T1/2= 9.59 h
T1/2= 6.81 h
T1/2= 8.25 h
T1/2= 5.14 h
Hours
L
-log10(pvalue)
PFKM
HK2
ENO1
GLUT1
PFKL
shNC VS shNSUN2
log2(Fold Change)
M
Spearman
R = 0.844
P < 0.001
m5C modification
Glucose metabolism
N
Spearman
R = 0.604
P < 0.001
NSUN2
Glucose metabolism
P
oeNC oeNSUN2
Glc. – + – +
NSUN2
GLUT1
HK2
PFKM
ENO1
PKM2
LDHA
β-actin
Q
Relative glucose uptake
*
oeNC oeNSUN2
% of Max
2-NBDG
R
ns
****
**
Relative glucose content of supernatants
2-DG – + – +
oeNC oeNSUN2
S
oeNC
oeNSUN2
Glucose
Oligomyci
2-DG
ECAR (mpH/min)
Time (minutes)
T
oeNC oeNSUN2
**
**
ECAR (mpH/min)
Glycolysis
Glycolytic Capacity
U
****
Relative concentration of pyruvic acid
oeNC oeNSUN2
V
**
Relative concentration of lactate
oeNC oeNSUN2
W
Huh7 cells
HCCLM3 cells
***
***
Relative ATP content
shNC shNSUN2 oeNC oeNSUN2

Fig. S4. NSUN2-mediated m^5C modification regulates metabolic reprogramming in a glucose-dependent manner, related to Fig. 4.

A and B. The expression levels of ADAR (**A**) and IGF2BP3 (**B**) in the murine TEM.

C and D. UMAP plot demonstrating the expression levels of ADAR (**C**) and IGF2BP3 (**D**) in the single-cell TEM.

E. Real-time qPCR analysis of Huh7 and HCCLM3 cells starved with glucose and restored with 5.5 mM glucose (n = 3 biological replicates).

F and G. Proliferation of shNC and shNSUN2 Huh7 cells (**F**), oeNC and oeNSUN2 HCCLM3 (**G**), evaluated by CCK-8 assay (n = 3 biological replicates).

H. Transwell migration assays assessing the migratory capacity of oeNC and oeNSUN2 HCCLM3 cells (n = 3 biological replicates).

I and J. Huh7 (**I**) and HCCLM3 (**J**) cells were glucose starved and restored with glucose, followed by dot blot assay of m^5C levels (n = 3 biological replicates).

K. HCCLM3 cells without or with glucose starvation for 4 hours and restored with 5.5 mM glucose 2 hours before dot blot assay of m^5C levels (global RNA) (n = 3 biological replicates).

L. The mRNAs exhibiting differential m^5C methylation levels ($p < 0.05$, $|\log_2(\text{Fold change})| > 2$) between shNC and shNSUN2 cells.

M and N. Correlation analysis between m^5C methylation GSVA enrichment scores (**M**), NSUN2 expression levels (**N**), and glucose metabolism GSVA enrichment scores in the TCGA-LIHC cohort.

O. RNA decay assay in shNC and shNSUN2 Huh7 cells treated with actinomycin D (Act.

D, 5 μg/mL), glucose starved and restored with glucose. Real-time qPCR against β-actin was performed to assess the half-life of PFKM mRNA (n = 3 biological replicates).

P. Immunoblotting analysis of oeNC and oeNSUN2 HCCLM3 cells after 6 hours of glucose starvation and subsequent 2 hours of restoration with 5.5 mM glucose.

Q. The glucose uptake capacity in oeNC and oeNSUN2 HCCLM3 cells, measured using flow cytometry to detect fluorescence intensity of 2-NBDG (n = 3 biological replicates).

R. Glucose content in the supernatant of oeNC and oeNSUN2 HCCLM3 cells after 48 hours of treatment with or without 5.5 mM 2-DG.

S and T. Measurements were recorded over time, with exposure to glucose, oligomycin, and 2-DG for ECAR assessment. ECAR in oeNC and oeNSUN2 HCCLM3 cells was recorded three times per condition (**S**). Glycolysis (ECAR following glucose addition) and glycolytic capacity (maximal ECAR after subtracting the ECAR following 2-DG exposure) were calculated (**T**).

U and V. Comparison of the relative pyruvic acid (**U**) and lactate (**V**) production between oeNC and oeNSUN2 HCCLM3 cells.

W. Comparison of relative ATP content between: shNC vs. shNSUN2 Huh7 cells, oeNC vs. oeNSUN2 HCCLM3 cells.

Data are mean ± SD. *p < 0.05, **p < 0.01, ***p < 0.001, ****p < 0.0001, ns, non-significant Student's t test (F-H, Q and T-W) or by two-way ANOVA (A-B, O and R).

Fig. S5

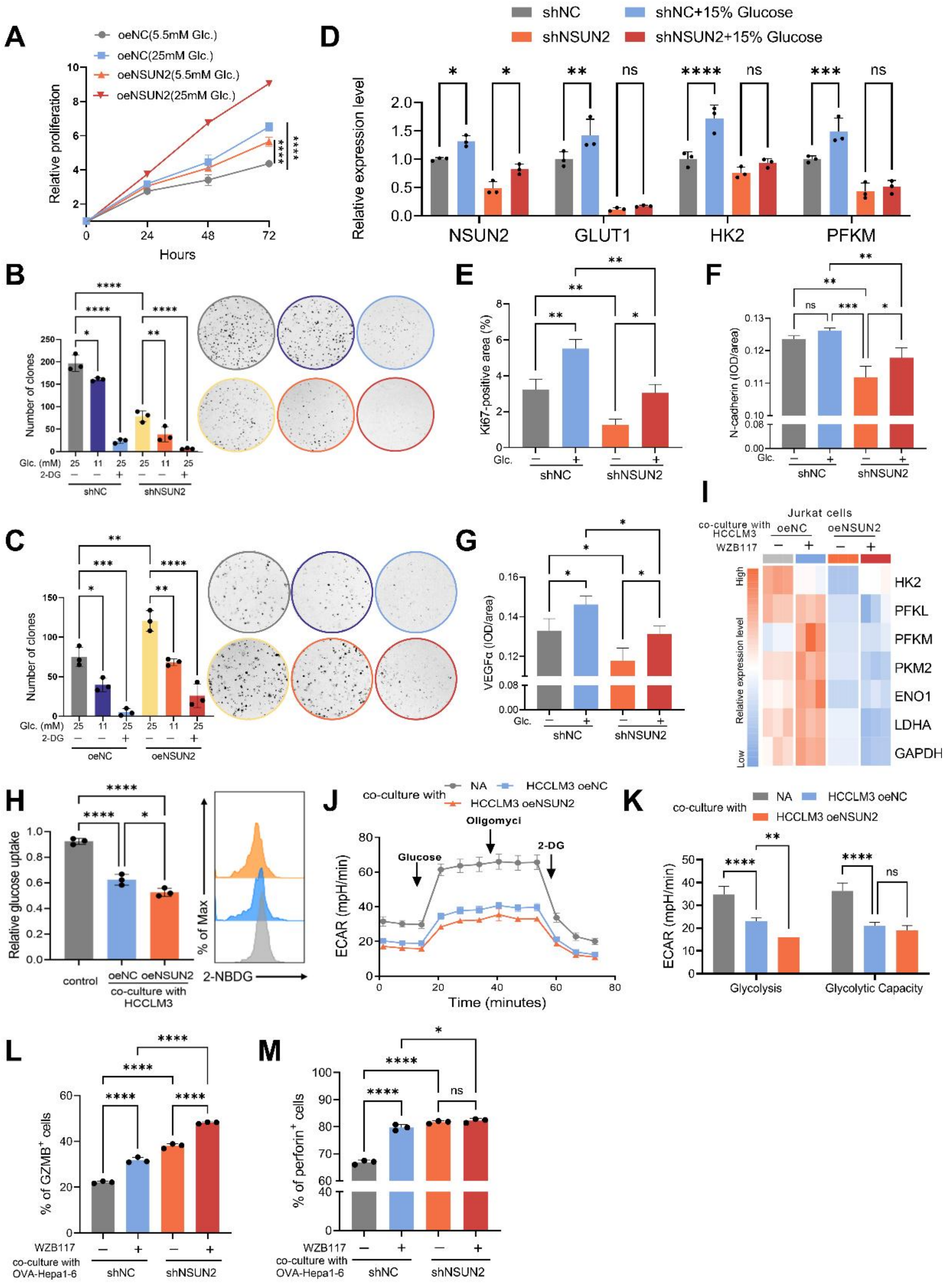
A
oeNC(5.5mM Glc.)
oeNC(25mM Glc.)
oeNSUN2(5.5mM Glc.)
oeNSUN2(25mM Glc.)
Relative proliferation
Hours
D
shNC
shNC+15% Glucose
shNSUN2
shNSUN2+15% Glucose
Relative expression level
NSUN2
GLUT1
HK2
PFKM
B
Number of clones
Glc. (mM)
2-DG
shNC
shNSUN2
E
Ki67-positive area (%)
Glc.
shNC
shNSUN2
F
N-cadherin (IOD/area)
Glc.
shNC
shNSUN2
C
Number of clones
Glc. (mM)
2-DG
oeNC
oeNSUN2
G
VEGFα (IOD/area)
Glc.
shNC
shNSUN2
I
Jurkat cells
co-culture with HCCLM3
oeNC
oeNSUN2
WZB117
High
Relative expression level
Low
HK2
PFKL
PFKM
PKM2
ENO1
LDHA
GAPDH
H
Relative glucose uptake
control
oeNC oeNSUN2
co-culture with HCCLM3
% of Max
2-NBDG
J
co-culture with
NA
HCCLM3 oeNC
HCCLM3 oeNSUN2
Oligomyci
Glucose
2-DG
ECAR (mpH/min)
Time (minutes)
K
co-culture with
NA
HCCLM3 oeNC
HCCLM3 oeNSUN2
ECAR (mpH/min)
Glycolysis
Glycolytic Capacity
L
% of GZMB+ cells
WZB117
co-culture with OVA-Hepa1-6
shNC
shNSUN2
M
% of perforin+ cells
WZB117
co-culture with OVA-Hepa1-6
shNC
shNSUN2

Fig. S5. The glucose-competition/NSUN2 axis drives tumor evolution and $CD8^+$ T cell dysfunction, related to Fig. 5.

A. Proliferation of oeNC and oeNSUN2 HCCLM3 cells, evaluated by CCK-8 (Cell Counting Kit-8) assay (n = 3 biological replicates). Cells were cultured in media containing 5.5 mM or 25 mM glucose.

B and C. Colony formation assays of shNC and shNSUN2 Huh7 cells (**B**), as well as oeNC and oeNSUN2 HCCLM3 cells (**C**), treated with 25 mM glucose, 11 mM glucose, or 5.5 mM 2-DG (n = 3 biological replicates).

D. Quantitative analysis of immunoblot signals for key glycolytic proteins shown in Fig. 5E. Protein levels were normalized to β-actin.

E-G. Quantification of Ki67-positive areas (**E**) was performed on the tumor samples as described in **Fig. 5F**. For N-cadherin (**F**) and VEGFα (**G**), the average optical density (IOD/Area) was measured to evaluate their expression levels.

H. Changes in glucose uptake capacity in Jurkat cells following 48 hours of co-culture with oeNC and oeNSUN2 HCCLM3 cells at a 1:1 ratio in 5.5 mM glucose medium. Glucose uptake was measured by flow cytometry based on the fluorescence intensity of 2-NBDG.

I. Following a 48 hours co-culture of Jurkat cells with oeNC or oeNSUN2 HCCLM3 cells at a 1:1 ratio in 5.5 mM glucose medium, we analyzed the expression levels of glycolysis-related genes within Jurkat cells. Co-culture was performed in medium supplemented with or without 10 μM WZB117. Gene expression levels were normalized to β-actin.

J and K. Following the co-culture of Jurkat cells with oeNC and oeNSUN2 HCCLM3 cells

at a 1:1 ratio for 48 hours in 5.5 mM glucose medium, we proceeded to measure the extracellular acidification rate (ECAR) of the Jurkat cells. Measurements were recorded over time, with exposure to glucose, oligomycin, and 2-DG for ECAR assessment. ECAR was recorded three times per condition (**J**). Glycolysis (ECAR following glucose addition) and glycolytic capacity (maximal ECAR after subtracting the ECAR following 2-DG exposure) were calculated (**K**).

L and M. OVA-specific TCR transgenic OT-1 $CD8^+$ T cells were isolated from the spleens of OT-1 mice and activated with the OVA-derived peptide SIINFEKL for five days. Subsequently, these activated OT-1 $CD8^+$ T cells were co-cultured with shNC or shNSUN2 OVA-Hepa1-6 cells at ratios of 1:1 for 48 hours in 5.5 mM glucose medium, in the presence or absence of 10 μM WZB117. The expression levels of GZMB and perforin in OT-1 $CD8^+$ T cells were analyzed by flow cytometry. Percentages of $GZMB^+$ (**L**) and $perforin^+$ cells (**M**) are shown as bar graphs.

Data are mean ± SD. *$p < 0.05$, **$p < 0.01$, ***$p < 0.001$, ****$p < 0.0001$, ns, non-significant by two-way ANOVA (A-H and J-M).

Supplementary Table 1. Evolutionary core genes (Only genes shared between homo sapiens and mus musculus were retained).

Cluster	Gene
**C1 & C4**	ABCD2, ABHD17C, AEBP2, AGER, AKAP9, APAF1, APLP2, ASXL1, AUH, BRCA2, BRD1, CAPN2, CAPRIN1, CEBPG, CHD7, CLTC, CRBN, CTNND1, CUEDC1, CYFIP1, DDX11, DGKD, EFCC1, ELF2, EPG5, EZH2, FUT8, GIGYF2, GOLGA2, GSTM5, ITPR1, JAK2, KCNN4, KLF9, KRR1, FAM84B, LTBP3, LTN1, MAN1C1, MSN, MSRB3, MT-RNR2, MYEF2, NAA30, NABP1, NACA, NF1, NNT, NPM2, NSRP1, OSBPL11, PAN3, PCMTD2, PLEKHH1, PPP2R2D, PROSER1, PRRC1, PRRC2A, PRSS22, PTAR1, PTPN3, RAB11FIP2, RAB22A, RALA, SEC24A, SEPT10, SGTB, SIAE, SIPA1L3, SLC16A4, SMCHD1, SMIM10L1, SNX30, SNX6, SPTAN1, SREK1IP1, SRSF3, SSH2, TJP1, TMEM161B, TMEM62, TNFRSF18, TPSB2, TRIM8, TRMT2B, USO1, VRK2, WDR48, WIF1, WIPF2, XPOT, ZFP41
**C3**	AAR2, ACTR5, BOLA1, LOH12CR1, CAPNS1, CCDC12, CCNF, CCNK, COQ6, DBNDD2, DDX41, DNAJC17, DPH7, EIF4H, ELOF1, EMC4, EPHA2, ESRP2, EXOC7, FBLIM1, FBXL8, FLOT2, GOT2, IRF2BP1, ITGA5, JUND, LHPP, LZIC, MED29, MOSPD3, MPZL3, MRPL32, MVB12A, MVK, MYBBP1A, NDUFA8, NDUFB7, NTMT1, NUDT16L1, NUDT18, NUP133, PES1, PGPEP1, PIAS4, PPM1M, PRPF19, QTRT1, RBBP4, RPS12, UTP23

Supplementary Table 2. Differentially m^5C-modified sites in GLUT1, HK2 and PFKM mRNAs: shNC versus shNSUN2 cells.

GeneName	GLUT1	GLUT1	GLUT1	HK2	PFKM
chrom	chr1	chr1	chr1	chr2	chr12
txStart	42958633	42929872	42931201	74833980	48122646
txEnd	42959080	42930035	42931206	74834643	48122859
PeakID	diffreps_ peak_3815	diffreps_ peak_3810	diffreps_ peak_3813	diffreps_ peak_64782	diffreps_ peak_24630
score	6.625661924	10.89549143	8.671948226	8.053292445	8.65942789
Peak_length	447	163	5	663	213
Foldchange	2.942979767	2448.5	467.8	3529	1075.5
P_value	2.36776E-07	1.27206E-11	2.12839E-09	8.84520E-09	2.19065E-09
FDR	8.37084E-07	3.19678E-10	1.59140E-08	4.42877E-08	1.59140E-08
Regulation	up	up	up	up	up

Supplementary Materials

Antibodies

Name	Supplier	Cat No.
**Rabbit anti-Ki-67 antibody**	Proteintech	Cat# 84432-1-RR
**Rabbit anti-N-cadherin antibody**	Proteintech	Cat# 22018-1-AP
**Rabbit anti-VEGFα antibody**	Proteintech	Cat# 19003-1-AP
**Rabbit anti-CD8 antibody**	Abcam	Cat# ab217344
**Rabbit anti-NSUN2 antibody**	Proteintech	Cat# 20854-1-AP
**Rabbit anti-5-Methylcytosine (5-mC) antibody**	Cell Signaling Technology	Cat# 28692
**Rabbit anti-GLUT1 antibody**	Proteintech	Cat# 21829-1-AP
**Rabbit anti-HK2 antibody**	Proteintech	Cat# 22029-1-AP
**Rabbit anti-PFKM antibody**	Proteintech	Cat# 55028-1-AP
**Mouse anti-ENO1 antibody**	Proteintech	Cat# 11204-1-AP
**Rabbit anti-PKM2 antibody**	Proteintech	Cat# 15822-1-AP
**Rabbit anti-LDHA antibody**	Proteintech	Cat# 19987-1-AP
**Mouse anti-β-actin antibody**	Proteintech	Cat# 66009-1-Ig
**PE anti-mouse CD45 antibody**	Biolegend	Cat# 147712
**FITC anti-mouse CD3 antibody**	Biolegend	Cat# 100204
**Brilliant Violet 510™ anti-mouse CD8a antibody**	Biolegend	Cat# 100752
**PerCP/Cyanine5.5 anti-mouse TNF-αantibody**	Biolegend	Cat# 506322
**Brilliant Violet 650™ anti-mouse IFN-γ antibody**	Biolegend	Cat# 505832
**Pacific Blue™ anti-human/mouse Granzyme B Recombinant antibody**	Biolegend	Cat# 372217
**APC anti-mouse Perforin antibody**	Biolegend	Cat# 154404
**Mouse anti-PD-L1 antibody**	Proteintech	Cat# 66248-1-Ig

Cell lines

Name	Supplier	Cat No.
**Human: Huh7**	JCRB	Cat# JCRB0403
**Human:**	Type Culture Collection of Chinese Academy of	Cat# SCSP-528

**HCCLM3**	Science	
**Human: Jurkat**	Type Culture Collection of Chinese Academy of Science	Cat# SCSP-513
**Mouse: Hepa1-6**	Type Culture Collection of Chinese Academy of Science	Cat# SCSP-512

Primers

Name	Sequence	Supplier
**NSUN2 qPCR primer-F**	CAAGCTGTTCGAGCACTACTAC	TsingKe
**NSUN2 qPCR primer-R**	CTCCCTGAGAGCGTCCATGA	TsingKe
**Nsun2 qPCR primer-F**	AGGTGGCTATCCCGAGATCG	TsingKe
**Nsun2 qPCR primer-R**	GACTCCATGAATTGGTCCCATT	TsingKe
**HK2 qPCR primer-F**	GAGCCACCACTCACCCTACT	TsingKe
**HK2 qPCR primer-R**	CCAGGCATTCGGCAATGTG	TsingKe
**PFKL qPCR primer-F**	GCTGGGCGGCACTATCATT	TsingKe
**PFKL qPCR primer-R**	TCAGGTGCGAGTAGGTCCG	TsingKe
**PFKM qPCR primer-F**	GGTGCCCGTGTCTTCTTTGT	TsingKe
**PFKM qPCR primer-R**	AAGCATCATCGAAACGCTCTC	TsingKe
**ENO1 qPCR primer-F**	AAAGCTGGTGCCGTTGAGAA	TsingKe
**ENO1 qPCR primer-R**	GGTTGTGGTAAACCTCTGCTC	TsingKe
**PKM2 qPCR primer-F**	ATGTCGAAGCCCCATAGTGAA	TsingKe
**PKM2 qPCR primer-R**	TGGGTGGTGAATCAATGTCCA	TsingKe
**GAPDH qPCR primer-F**	TGTGGGCATCAATGGATTTGG	TsingKe
**GAPDH qPCR primer-R**	ACACCATGTATTCCGGGTCAAT	TsingKe
**TPI1 qPCR primer-F**	CTCATCGGCACTCTGAACG	TsingKe
**TPI1 qPCR primer-R**	GCGAAGTCGATATAGGCAGTAGG	TsingKe

**LDHA qPCR primer-F**	AGGAGAAACACGCCTTGATTTAG	TsingKe
**LDHA qPCR primer-R**	ACGAGCAGAGTCCAGATTACAA	TsingKe
**ACTB qPCR primer-F**	ACCGGGCATAGTGGTTGGA	TsingKe
**ACTB qPCR primer-R**	ATGGTACACGGTTCTCAACATC	TsingKe
**Actb qPCR primer-F**	GGCTGTATTCCCCTCCATCG	TsingKe
**Actb qPCR primer-R**	CCAGTTGGTAACAATGCCATGT	TsingKe
**Hk2 qPCR primer-F**	ATGATCGCCTGCTTATTCACG	TsingKe
**Hk2 qPCR primer-R**	CGCCTAGAAATCTCCAGAAGGG	TsingKe
**Pfkl qPCR primer-F**	GGAGGCGAGAACATCAAGCC	TsingKe
**Pfkl qPCR primer-R**	GCACTGCCAATAATGGTGCC	TsingKe
**Pfkm qPCR primer-F**	CATCGCCGTGTTGACCTCT	TsingKe
**Pfkm qPCR primer-R**	CCCGTGAAGATACCAACTCGG	TsingKe
**Eno1 qPCR primer-F**	TGCGTCCACTGGCATCTAC	TsingKe
**Eno1 qPCR primer-R**	CAGAGCAGGCGCAATAGTTTTA	TsingKe
**Pkm2 qPCR primer-F**	CGCCTGGACATTGACTCTG	TsingKe
**Pkm2 qPCR primer-R**	GAAATTCAGCCGAGCCACATT	TsingKe
**Gapdh qPCR primer-F**	AATGGATTTGGACGCATTGGT	TsingKe
**Gapdh qPCR primer-R**	TTTGCACTGGTACGTGTTGAT	TsingKe
**Tpi1 qPCR primer-F**	GAGAGAGCCGTGCGTTTGTA	TsingKe
**Tpi1 qPCR primer-R**	CCCCAACGAAGAACTTCCTGG	TsingKe
**Ldha qPCR primer-F**	GGGCTACAAGCATCTTGAGAG	TsingKe
**Ldha qPCR primer-R**	GACACGTTGCACCTGACTG	TsingKe

Plasmids

Name	Supplier	Cat No.
**pCDH-CMV-MCS-EF1-Puro**	Youbio	Cat# VT1480
**pCDH-Puro-NSUN2**	This paper	NA
**pCDH-Puro-Nsun2**	This paper	NA
**pLVX-Puro**	Youbio	Cat# VT1465
**pLVX-Puro-OVA**	This paper	NA

Biological samples

Description	Source
**Human HCC tumor tissue (n=10)**	Sir Run Run Shaw Hospital, Zhejiang University

Reagents and kits

Description	Source	Identifier
**EZ-press RNA Purification Kit**	EZBioscience	Cat# B0004DP
**MagiSort Mouse CD8$^+$ T cell Isolation Kit**	Thermo Fisher	Cat# 8804682274
**CellTrace CFSE Cell Proliferation Kit**	Thermo Fisher	Cat# C34570
**PI/RNase Staining Buffer**	BD	Cat# 550825
**Recombinant Murine IL-2**	Peprotech	Cat# 212-12
**Recombinant Human IL-2**	Peprotech	Cat# 200-02
**Fixable Viability Stain 780**	BD	Cat# 565388
**Intracellular Staining Perm Wash Buffer (10X)**	Biolegend	Cat# 421002
**Fixation Buffer**	Biolegend	Cat# 420801
**Actinomycin D**	MedChemExpress	Cat# HY-17559
**Protein A+G Agarose**	Beyotime	Cat# P2055
**Seahorse XFe96/XF Pro FluxPak Mini**	Anligent	Cat# 103793-100
**β - Hydroxybutanol**	Sigma-Aldrich	Cat# M3148-25ML
**Cell counting kit (CCK-8)**	Yeasen Biotech	Cat# 40203ES60
**Endotoxin-free plasmid purification kit**	Tiangen	Cat# DP117
**D-(+)-Glucose**	Beyotime	Cat# ST1228-1kg
**2-NBDG**	MedChemExpress	Cat# HY-116215
**2-Deoxy-D-glucose**	MedChemExpress	Cat# HY-13966
**WZB117**	MedChemExpress	Cat# HY-19331

**OVA G4 peptide**	MedChemExpress	Cat# HY-P1771
**BAY-876**	MedChemExpress	Cat# HY-100017
**Methylene Blue Solution，0.2%**	Solarbio	Cat# G1301
**L-Lactic Acid (LA) Colorimetric Assay Kit**	Elabscience	Cat# E-BC-K044-S
**Pyruvic Acid Colorimetric Assay Kit**	Elabscience	Cat# E-BC-K130-M
**Glucose (GLU) Fluorometric Assay Kit**	Elabscience	Cat# E-BC-F037
**RNase Inhibitor, recombinant (human placenta)**	New England Biolabs	Cat# M0307S
**WB/IP lysis buffer**	Beyotime	Cat# P0013J
**TRIzol**	TAKARA	Cat# 15596026
**Protease inhibitor cocktail**	Abcam	Cat# ab65621
**Lipofectamine 3000 reagent**	Invitrogen	Cat# L3000015
**BCA protein assay kit**	Invitrogen	Cat# M34152
**Anti-Mouse PD-L1/B7-H1 Antibody (10F.9G2)**	MedChemExpress	Cat# HY-P99145
**ImmunoCult™ Human CD3/CD28 T Cell Activator**	STEMCELL	Cat# 100-0784
**Dynabeads™ Mouse T-Activator CD3/CD28**	Gibco	Cat# 11456D
